# Visual motion and decision-making in dyslexia: Reduced accumulation of sensory evidence and related neural dynamics

**DOI:** 10.1523/JNEUROSCI.1232-21.2021

**Published:** 2021-11-15

**Authors:** Catherine Manning, Cameron D. Hassall, Laurence, T. Hunt, Anthony M. Norcia, Eric-Jan Wagenmakers, Margaret J. Snowling, Gaia Scerif, Nathan J. Evans

**Affiliations:** a Department of Experimental Psychology, University of Oxford, UK; b School of Psychology and Clinical Language Sciences, University of Reading, UK; c Department of Psychiatry, University of Oxford, UK; d Department of Psychology, Stanford University, USA; e Faculty of Social and Behavioural Sciences, University of Amsterdam, The Netherlands; f School of Psychology, University of Queensland, Australia

## Abstract

Children with and without dyslexia differ in their behavioural responses to visual information, particularly when required to pool dynamic signals over space and time. Importantly, multiple processes contribute to behavioural responses. Here we investigated which processing stages are affected in children with dyslexia when performing visual motion processing tasks, by combining two methods that are sensitive to the dynamic processes leading to responses. We used a diffusion model which decomposes response time and accuracy into distinct cognitive constructs, and high-density EEG. 50 children with dyslexia (24 male) and 50 typically developing children (28 male) aged 6 to 14 years judged the direction of motion as quickly and accurately as possible in two global motion tasks (motion coherence and direction integration), which varied in their requirements for noise exclusion. Following our pre-registered analyses, we fitted hierarchical Bayesian diffusion models to the data, blinded to group membership. Unblinding revealed reduced evidence accumulation in children with dyslexia compared to typical children for both tasks. Additionally, we identified a response-locked EEG component which was maximal over centro-parietal electrodes which indicated a neural correlate of reduced drift-rate in dyslexia in the motion coherence task, thereby linking brain and behaviour. We suggest that children with dyslexia tend to be slower to extract sensory evidence from global motion displays, regardless of whether noise exclusion is required, thus furthering our understanding of atypical perceptual decision-making processes in dyslexia.

## Introduction

It has long been suspected that visual processing relates to the reading difficulties characterising developmental dyslexia (e.g., Hinshelwood, 1896; Lovegrove et al., 1980). One visual function that develops atypically in those with dyslexia is visual motion processing: an important ability contributing to scene segmentation, depth perception and object recognition (Braddick et al., 2003). Difficulties in global motion tasks requiring integration over space and time have been widely reported in dyslexia (Benassi et al., 2010). Typically, participants are required to detect or discriminate coherently moving signal dots amongst randomly moving noise dots (Newsome & Paré, 1988). In this ‘motion coherence’ task, dyslexic individuals tend to have elevated psychophysical thresholds, requiring higher proportions of signal dots to perform at the same level of accuracy as those without dyslexia (Benassi et al. 2010). The nature of the relationship is still being debated, with some researchers proposing a causal relationship between motion sensitivity and reading ability (Boets et al., 2011; Gori et al., 2016; but see Goswami, 2015; Joo et al., 2017; Olulade et al., 2013; Piotrowska & Willis, 2019).

Atypical global motion processing in dyslexia may reflect reduced sensitivity to rapid temporal information originating from deficiencies in the magnocellular system (Livingstone et al., 1991; Stein, 2001, 2019; Stein & Walsh, 1997) or related dorsal stream (Braddick et al., 2003; Hansen et al., 2001), which are particularly specialised for motion perception (Livingstone & Hubel, 1988). Alternative accounts suggest that dyslexic individuals have difficulty filtering out the randomly moving noise dots in motion coherence tasks (“noise exclusion”; Conlon et al., 2012; Sperling et al., 2006) or difficulties integrating over space and time (Benassi et al., 2010; Hill & Raymond, 2002; Raymond & Sorensen, 1998).

**Figure 1:**
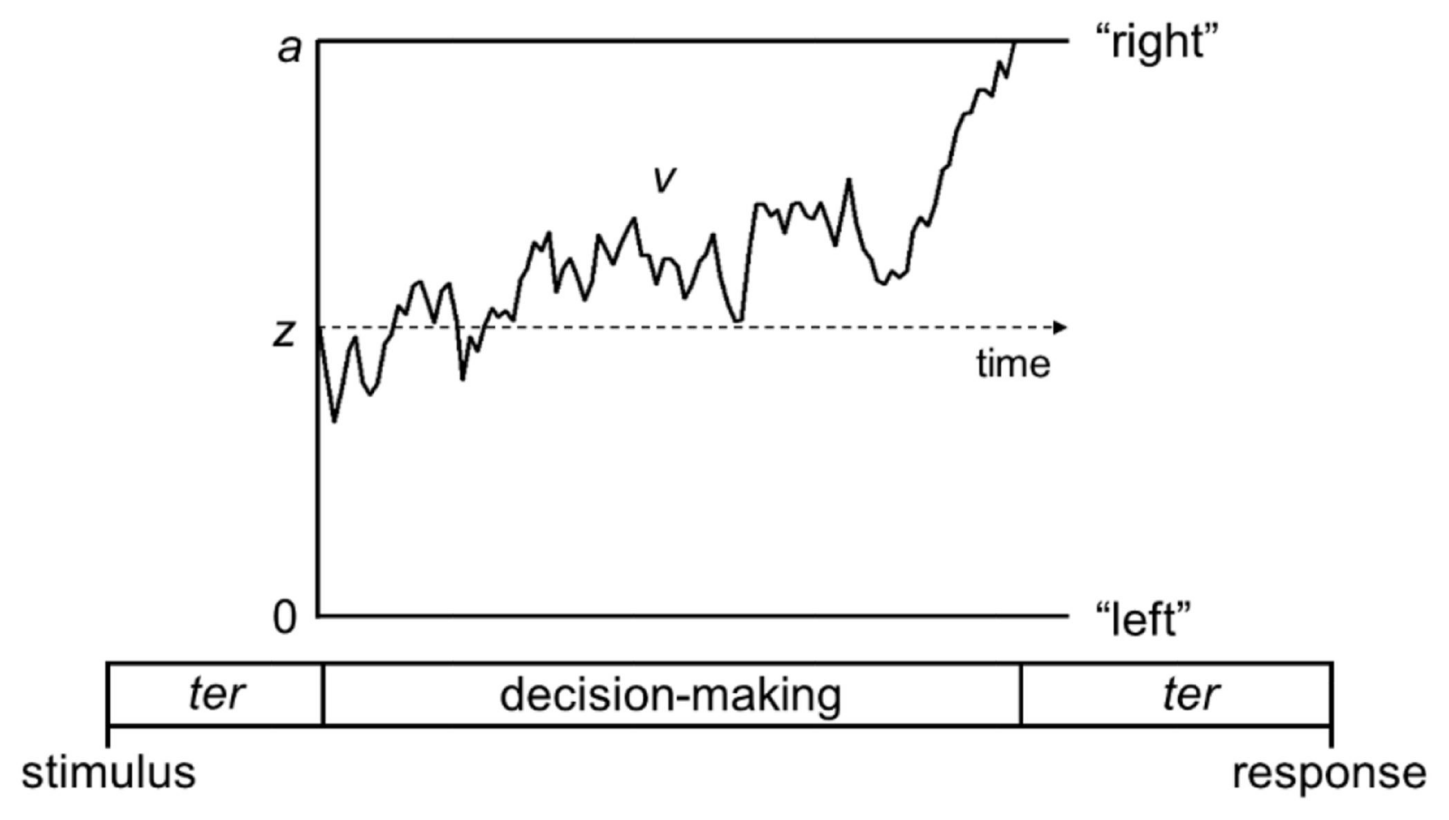
Schematic representation of the decision-making process in the diffusion model for a trial with rightward motion Decision-making process represented as a noisy accumulation of evidence from a starting point, *z*, towards one of two decision bounds. In our motion tasks, the decision bounds correspond to left and right responses. Boundary separation, *a*, represents the width between the two bounds and reflects response caution. Wider decision boundaries reflect that more evidence is required before making a decision (i.e., more cautious responses). Drift-rate, *v*, reflects the rate of evidence accumulation, which depends on both the individual’s sensitivity to a stimulus and the stimulus strength. Non-decision time, *ter*, is the time taken for sensory encoding processes prior to the decision-making process and response generation processes after a bound is reached.

Despite focusing on the sensory parameters of visual motion stimuli, these accounts give little consideration to the dynamic processes leading to atypical behavioural responses in dyslexia, and particularly, whether decision-making processes are affected. Here we explicitly modelled the decision-making process using a popular cognitive model of accuracy and response time: the diffusion model (Evans & Wagenmakers, 2020; Ratcliff, 1978; Stone, 1960). The decision is modelled as a noisy evidence accumulation process from a starting point towards one of two decision bounds (Figure 1). This modelling approach will help identify the locus of atypical processing in dyslexia, with two further advantages. First, the resulting parameters may be more sensitive to group differences than accuracy or response time alone (Stafford et al., 2020) and second, the parameters relate well to neural measures (Kelly & O’Connell, 2013; Manning et al., 2021a; Turner et al., 2015). Accordingly, we combined the diffusion model with a neural measure sensitive to the dynamic processes contributing to behavioural responses (EEG), bridging brain and behaviour.

The diffusion model was recently applied to motion coherence performance in children with varying reading abilities (O’Brien and Yeatman, 2020). Poorer reading was related to lower drift-rates, wider decision bounds, and more intra-individual variability in starting point and non-decision time. Therefore poor readers accumulated motion evidence more slowly and responded more cautiously than good readers.

Here, we used diffusion models to identify the processing stages affected in children with dyslexia across two global motion tasks. The first task was a standard motion coherence task (cf. O’Brien & Yeatman, 2020). The second task was a direction integration task not used before with dyslexic individuals, whereby dot directions are sampled from a Gaussian distribution, with difficulty manipulated via the standard deviation of the distribution. In this task, the optimal strategy is to average over all dots, with no noise exclusion requirement. The reason for presenting both tasks to children with dyslexia was to determine whether differences in model parameters are found for both motion tasks, suggesting a general motion-processing deficit (cf. magnocellular/dorsal deficit; Braddick et al., 2003; Stein, 2001), or whether differences in model parameters are found particularly for the motion coherence task, reflecting noise exclusion difficulties (Conlon et al., 2012; Sperling et al., 2006).

## Methods

### Pre-registration

We pre-registered our inclusion criteria and analysis plan before completing data collection and before commencing analyses (https://osf.io/enkwm). When analysing the data we used a blind modelling approach to ensure that modelling decisions were not biased by our hypotheses. Our pre-registered primary research questions and hypotheses were:

1. *Do children with dyslexia have reduced drift-rates in a motion coherence task compared to typically developing children?* We hypothesised that children with dyslexia would have reduced drift-rates in the motion coherence task compared to typically developing children, in line with the results of O’Brien and Yeatman (2020) and reports of reduced motion coherence sensitivity in dyslexic individuals (Benassi et al., 2010).
2. *Do children with dyslexia have reduced drift-rates in a direction integration task compared to typically developing children?* If children with dyslexia show difficulties with all global motion tasks (in line with impaired magnocellular/dorsal stream functioning; Braddick et al., 2003; Stein, 2001), then we would expect children with dyslexia to have a reduced drift-rate in this task as well. Instead, if the performance of children with dyslexia in a motion coherence task is limited solely by difficulties with noise exclusion (Conlon et al., 2012; Sperling et al., 2006), we would expect to see no difference between children with and without dyslexia in this task, as it does not require segregating signal dots from randomly moving noise dots.
3. *Do children with dyslexia show increased boundary separation?* We hypothesised that children with dyslexia would have wider boundary separation compared to typically developing children in both tasks, following O’Brien and Yeatman (2020).
4. *Do children with dyslexia show increased non-decision time?* We hypothesised no group differences in overall non-decision time in either task, following O’Brien and Yeatman (2020).

### Participants

We collected data from 50 children with dyslexia and 60 typically developing children who met our inclusion criteria. Specifically, participants were required to be aged 6 to 14 years (inclusive), have verbal and/or performance IQ scores above 70 (measured using the Wechsler Abbreviated Scales of Intelligence, 2^nd^ edition [WASI-2]; Wechsler, 2011) and to have normal or corrected-to-normal acuity, as measured using a Snellen acuity chart (with binocular acuities of 6/9 or better for children aged 6 to 8 years and 6/6 or better for children aged 9 to 14 years). Children in the dyslexia group were required to have a dyslexia diagnosis (or be in the process of obtaining one, *n* = 1), and to have a reading and spelling composite score of 89 or below, which was computed by averaging the standard scores for the spelling subtest of the Wechsler Individual Achievement Test (WIAT-III; Wechsler, 2017) and the Phonological Decoding Efficiency subtest of the Test of Word Reading Efficiency (TOWRE-2; Torgesen et al., 2012). A cut-off of 89 was chosen to correspond to 1.5 standard deviations below the mean of typically developing children in a similar study (Snowling et al., 2019a, 2019b). Children in the typically developing group were required to have composite scores above 89 and to have no diagnosed developmental conditions. Datasets from an additional 4 typically developing children were excluded due to poor visual acuity (*n* = 1), having a composite score of 89 or below (*n* = 2), or failing to pass criterion on the task (*n* = 1), and datasets from an additional 11 children with dyslexia were excluded due to poor visual acuity (n = 2) or having a composite score above 89 (n = 9).

We then selected 50 typically developing children to best match the children with dyslexia in terms of age and performance IQ using the R MatchIt package (Ho et al., 2011), so that the final dataset included 50 children with dyslexia (24 male) and 50 typically developing children (28 male). As shown in Table 1, the children with dyslexia had slightly higher ages and lower IQ values on average than the typically developing children. EEG data were collected during task performance in 47 typically developing and 44 children with dyslexia (although EEG data were available only in the motion coherence task for one child with dyslexia and one typically developing child). The EEG data from these participants were included in a paper investigating responses locked to the onset of coherent motion in typically developing children and children with autism or dyslexia (Toffoli et al., 2021), and the larger group of 60 typically developing children were used to form the comparison group in an autism study (Manning et al., 2021b).

### Apparatus

The tasks were presented on a Dell Precision M3800 laptop (2048 x 1152 pixels, 60 Hz) using the Psychophysics Toolbox for MATLAB (Brainard, 1997; Kleiner, Brainard & Pelli, 2007; Pelli, 1997). EEG signals were collected using 128-channel Hydrocel Geodesic Sensor Nets connected to Net Amps 300 (Electrical Geodesics Inc., OR, USA) and NetStation 4.5 software. A photodiode attached to the monitor independently verified stimulus presentation timing. Participants used a Cedrus RB-540 response box (Cedrus, CA, USA).

### Stimuli

**Figure 2:**
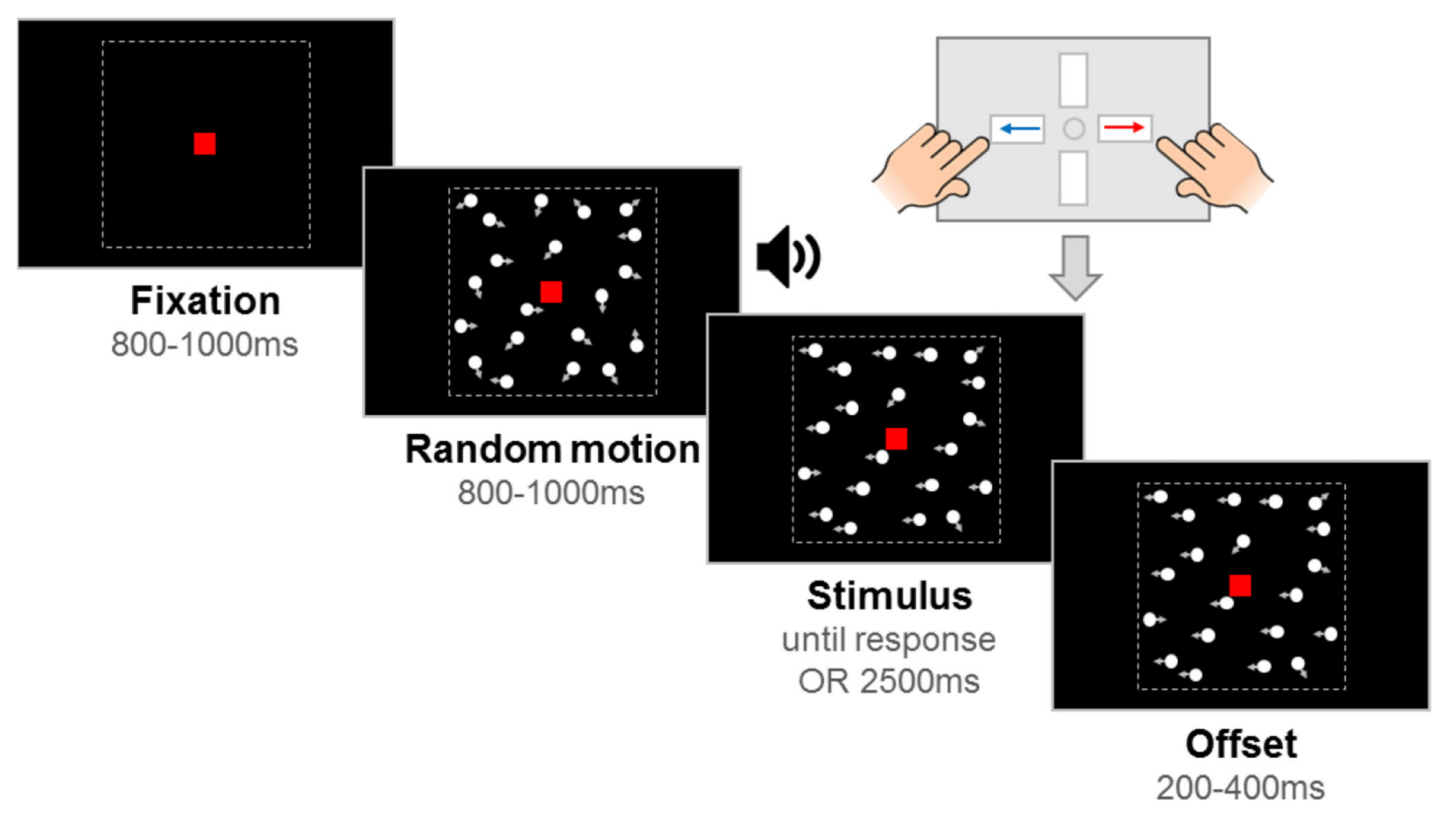
Schematic representation of trial procedure. The trial started with an initial *fixation* period that was followed by a *random motion* period consisting of random, incoherent moving dots, which was in turn followed by a *stimulus* containing leftward or rightward global motion. The child was asked to report the direction using a response box. After the response or after the maximum stimulus duration elapsed (2500 ms), the stimulus remained on the screen for a short *offset* period. Note that arrows (indicating movement) and dotted lines (marking the square stimulus region) are presented for illustration only. The stimulus shown here is from the motion coherence task, where a proportion of dots move coherently. In the direction integration task, dot directions were taken from a Gaussian distribution. Figure reproduced from https://osf.io/wmtpx/ under a CC-BY4.0 license.

Stimuli were 100 white, randomly positioned dots (diameter 0.19˚) moving at 6˚/s within a square aperture (10˚ x 10˚) on a black background, with a limited lifetime of 400 ms. Each trial had a fixation period, a random motion period, a stimulus period, and an offset period, with a red fixation square (0.24˚ x 0.24˚) presented throughout (see Figure 2). By presenting random (incoherent) motion before the stimulus period, we could dissociate evoked responses to directional motion from pattern- and motion-onset evoked potentials. The start of the stimulus period was highlighted to participants with an auditory tone. In the motion coherence task, directional motion (leftward or rightward) was introduced in a proportion of ‘signal’ dots, while the remainder of the dots continued to move in random directions. In the direction integration task, the directions of dots in the stimulus phase were distributed according to a Gaussian distribution with a mean leftward or rightward direction. The fixation period, random motion period and offset period had jittered durations within a fixed range, while the stimulus period was presented until a response or 2500 ms had elapsed. The offset period continued the directional motion to temporally separate motion offset from the response.

### Experimental task procedure

Children completed motion coherence and direction integration tasks within child-friendly games (based on Manning et al., 2019, 2021a). Using animations, participants were told that fireflies were escaping from their viewing boxes, and they were asked to tell the zookeeper which way the fireflies were escaping. There were 10 ‘levels’ of the game. Levels 1-5 corresponded to one task (either motion coherence or direction integration), and Levels 6-10 corresponded to the other task, with the order of tasks being counterbalanced across participants. Levels 1 and 6 were practice phases, and the remaining 4 levels for each task were experimental blocks. In the motion coherence task, difficulty was manipulated by varying the proportion of coherently moving dots, and in the direction integration task, difficulty was manipulated by varying the standard deviation of the Gaussian distribution from which the dot directions were sampled.

In the practice phases, four demonstration trials were presented with no random motion phase and an unlimited stimulus phase, so that the experimenter could explain the task. Participants reported stimulus direction using a response box. The first two demonstration trials were ‘easy’ (100% coherence or 1˚ standard deviation), and the last two were more difficult (75% and 50% coherence, or 10˚ and 25˚ standard deviations). Following the demonstration trials, there were up to 20 criterion trials with a coherence of 95% or a standard deviation of 5˚. These trials introduced the random motion phase. Participants were told that the fireflies would be going “all over the place” at first, and that they must wait for an alarm (auditory beep) before deciding which way the fireflies were escaping. A time limit was enforced, with visual feedback presented on the screen if participants did not respond within 2500 ms (“Timeout! Try to be quicker next time!”). Feedback on accuracy was given for responses made within the time limit (“That was correct!”, or “It was the other way that time”). When participants met a criterion of four consecutive correct responses, no more criterion trials were presented. Next, there were eight practice trials of increasing difficulty (motion coherence task: 80%, 70%, 60%, 50%, 40%, 30%, 20%, 10%; direction integration task: 5˚, 10˚, 15˚, 20˚, 30˚, 40˚, 50˚, 60˚) with feedback as before. Level 1 was repeated for one typically developing child and 2 children with dyslexia who did not meet the criterion of four consecutive correct responses on the first attempt, but passed on the second attempt.

Levels 2-5 and 7-10 each contained 38 trials, with 9 repetitions of each of two difficulty levels (motion coherence task: 30%, 75%; direction integration task: 70˚, 30˚ SD), for each motion direction (leftward, rightward), and an additional 2 catch trials presenting 100% coherent (0˚ SD) motion. The experimental phase for each task therefore consisted of 152 trials. No trial-by-trial feedback was presented during the experimental phase, apart from a ‘timeout’ message if no response was made within 2500ms after stimulus onset. At the end of each level, participants were given points for their speed and accuracy in the preceding block (computed by (1 / median response time) * the number of correct responses * 2, rounded to the nearest integer). If participants obtained a score under 10, a score of 10 points was given to maintain motivation. Trials were presented automatically, although the experimenter could pause and resume trial presentation if necessary. The experimental code can be found here: https://osf.io/fkjt6/.

### General procedure

The procedure was approved by the Central University Research Ethics Committee at the University of Oxford. Parents provided written informed consent and children gave verbal or written assent. All children took part at the University of Oxford apart from one child with dyslexia who was seen at school without EEG. During the experimental tasks, participants sat 80cm away from the computer screen in a dimly lit room. For children who participated with EEG, we fitted the net prior to the experiment and ensured that electrode impedances were below 50 kΩ. EEG data were acquired at a sampling rate of 500Hz with a vertex reference electrode.

Children were closely monitored by an experimenter sitting beside them. The experimenter provided general encouragement and task reminders, pausing before the start of a trial if needed (e.g., to remind the child to keep still). Children had short breaks at the end of each ‘level’ and a longer break at the end of the first task (at the end of ‘level 5’). During the longer break, electrode impedances were re-assessed for children wearing EEG nets. Children marked their progress through the levels using a stamper on a record card. The children also completed a Snellen acuity test, the WASI-2, the TOWRE-2 and the spelling subtest of the WIAT-III. The whole session took no longer than 2 hours and children were given a gift voucher to thank them for their time.

### Diffusion model analysis

Initially, a blinded analysis was conducted to ensure that modelling decisions were made without being biased by the hypotheses under test. The first author (CM) prepared a blinded dataset in which group membership was randomly permuted (see also Dutilh et al., 2017) and one of the authors (NJE) ran diffusion model analysis on this blinded dataset.

Prior to modelling, trials with response times under 200 ms were removed (corresponding to 0.20% of trials in the typical group and 0.24% of trials in the dyslexia group). Trials without a response (i.e., no response made within the 2500ms deadline) were modelled as non-terminating accumulation trajectories, with the probability of a non-response occurring being the survivor function for the model at the time of the 2500 ms deadline (Evans et al., 2018; Howard et al., 2020; Ulrich & Miller, 1994). These trials accounted for 1.02% of the data in the typical group and 1.26% of the data in the dyslexia group. We fit the data from each task with hierarchical, Bayesian diffusion models with 5 parameters: 1) average drift-rate across difficulty levels *v.mean*, 2) boundary separation *a*, 3) non-decision time *ter*, 4) difference in mean drift-rate between difficulty levels *v*.*diff*, and 5) starting point *z*. The stochastic noise within the model (*s*) was fixed at 0.1 to solve a scaling problem within the model, as per convention (Ratcliff, 1978). There were 3 hyperparameters for each parameter reflecting the mean (μ) and standard deviation (σ) across the two groups and the difference between groups (δ). Importantly, this parameterization allowed us to explicitly set priors on the differences between groups, which was the key effect of interest within the current study. More specifically, the priors were:

*Data level:*
$$y_{pi}∼diffusion\left(a_{p,}z_{p},{\text{Ter}}_{p},v_{pi},s\right)$$
*Parameters:*
$$\begin{array}{c} a_{p}∼N_{+}\left({\text{μ}}_{a}\pm \delta_{a},\sigma_{a}\right)\\ z_{p}/a_{p}∼TN_{0,1}\left({\text{μ}}_{z}\pm \delta_{z},\sigma_{z}\right)\\ Ter_{p}∼N_{+}\left({\text{μ}}_{Ter}\pm \delta_{Ter},\sigma_{Ter}\right)\\ v_{p1}-v_{p2}∼N\left({\text{μ}}_{v.diff}\pm \delta_{v.diff},\sigma_{v.diff}\right)\\ \frac{v_{p1}+v_{p2}}{2}∼N\left({\text{μ}}_{v.mean}\pm \delta_{v.mean},\sigma_{v.mean}\right)\\ s=0.1 \end{array}$$
*Hyperparameters:*
$$\begin{array}{c} \mu_{a}∼N_{+}(0.2,0.2)\\ \mu_{z}∼TN_{0,1}(0.5,0.2)\\ \mu_{Ter}∼N_{+}(0.3,0.3)\\ \mu_{v.diff}∼N(0,0.1)\\ \mu_{v.\text{mean}}∼N(0.3,0.3)\\ \sigma_{a},\sigma_{z},\sigma_{\text{Ter}},\sigma_{v,\text{diff}},\sigma_{v.\text{mean}}∼\text{Γ}(1,1)\\ \delta_{a},\delta_{z},\delta_{\text{Ter}},\delta_{\text{v}.\text{diff}},\delta_{\text{v}.\text{mean}}∼N(0,0.01) \end{array}$$

where *y* reflects the data, and subscripts *p* and *i* reflect the participant and difficulty level respectively. The priors for the *μ* and *σ* parameters were based on those used in previous studies implementing hierarchical diffusion models (e.g., Evans & Brown, 2017; Evans & Hawkins, 2019; Evans et al., 2019), and the priors for the δ parameters were based on the “moderately informative priors” used for the differences between conditions in Evans (2019). We used a differential evolution Markov chain Monte Carlo algorithm (DE-MCMC; Ter Braak, 2006; Turner, Sederberg, Brown, & Steyvers, 2013) to sample from the posterior with 15 interacting chains, each with 4000 iterations, the first 1500 of which were discarded as burn-in. We also implemented a migration algorithm (see Turner, Sederberg, Brown, & Steyvers, 2013), where chains were randomly migrated every 14 iterations between iterations 500 and 1100. We calculated Bayes factors through the Savage-Dickey ratio. Where we found evidence of group differences, we established the population effect size by dividing the posterior of the group difference (δ) by the posterior of the population standard deviation (σ).

As shown in Table 1, the children with dyslexia were on average slightly older and of lower IQ than the typically developing children. As pre-registered, the first author (CM) ran a default Bayesian t-test using the BayesFactor R package (Morey & Rouder, 2018) which revealed weak, inconclusive evidence for the absence of group differences in age (BF in support of group differences = 0.33; Jeffreys, 1961). As we know that diffusion model parameters change with age (Manning et al., 2021a), and as we couldn’t conclusively rule out group differences in age, we also ran models which partialled out the effects of age from all of the parameters (using the residuals from the line of best fit between age and each of the parameters), in addition to our standard models. In our pre-registered analysis plan we decided not to control for performance IQ as it may relate to both group membership and decision-making in cognitively relevant ways (Dennis et al., 2009). The analysis files were posted on the Open Science Framework prior to unblinding (https://osf.io/nvwf7/), at which point all models were re-run on the unblinded dataset with correct group membership.

### EEG analysis for joint modelling

We ran exploratory analysis on the unblinded dataset to investigate links between drift-rate and EEG activity. EEG data were band-pass filtered between 0.3 and 40 Hz in NetStation and then exported for further processing in MATLAB using EEGLAB functions (Delorme & Makeig, 2004). We downsampled each participant’s data to 250 Hz and selected only the data between the first fixation onset and the last offset period. We then bandpass-filtered between 0.3 and 40 Hz (due to insufficient attenuation of low frequencies by NetStation filters, Manning et al., 2019) and used EEGLAB’s ‘clean_artifacts’ function to remove bad channels, identify data segments with standard deviations over 15 and correct them using artifact subspace reconstruction (ASR; Chang et al., 2018). Missing channels were then interpolated. We then ran independent components analysis on 3000 ms epochs starting at fixation onset using an Infomax algorithm and subtracted ocular components from the continuous data. Finally, we average re-referenced the data. In line with the behavioural analyses, we excluded triggers for response events made <200 ms or >2500 ms after stimulus onset.

**Figure 3:**
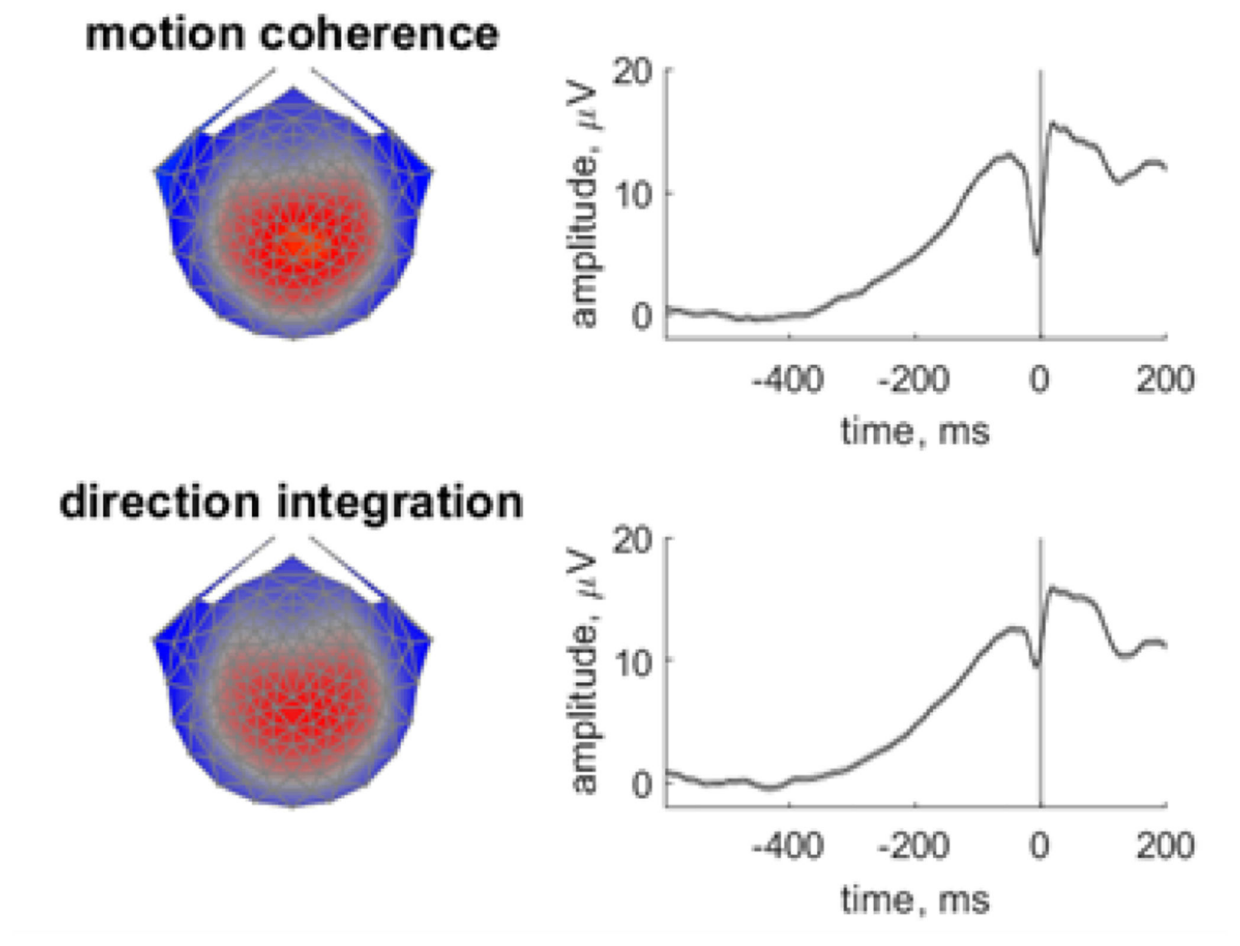
Scalp topographies and temporal dynamics for the most reliable component in the motion coherence and direction integration tasks Topographic visualisations of the forward-model projections of the most reliable component (left) reflecting the weights given to each electrode following reliable components analysis (RCA) on data from all participants pooled across difficulty level, for the motion coherence task (upper) and direction integration task (lower). The waveforms (right) show the temporal dynamics of the component.

Following previous work, we used a data-driven component decomposition technique to identify spatiotemporally reliable patterns of activity across trials, which has the effect of maximising signal-to-noise ratio (Reliable Components Analysis, Dmochowski et al., 2012; Dmochowski & Norcia, 2015; Manning et al., 2019, 2021a). To do this, we epoched each participant’s preprocessed continuous data from -600 ms to 200 ms around each response, and we baselined the data to the last 100 ms of the random motion period. We submitted the baselined epochs for participants in both groups to Reliable Components analysis for each task separately. The forward-model projections of the weights for the most reliable component for each task (which explained 28.7% and 27.1% of the reliability in the motion coherence and direction integration tasks, respectively) are shown in Figure 3. This component resembled the most reliable component found in our previous work (Manning et al., 2021a), which in turn resembles the centro-parietal positivity (O’Connell et al., 2012; Kelly and O’Connell, 2013). Build-up of activity in this component has been linked to drif-trate in typically developing children (Manning et al., 2021a). To investigate links with drif-trate in the current dataset, we projected each participant’s continuous data through the spatial weights for this component to yield a single component waveform for each participant for each task.

**Figure 4:**
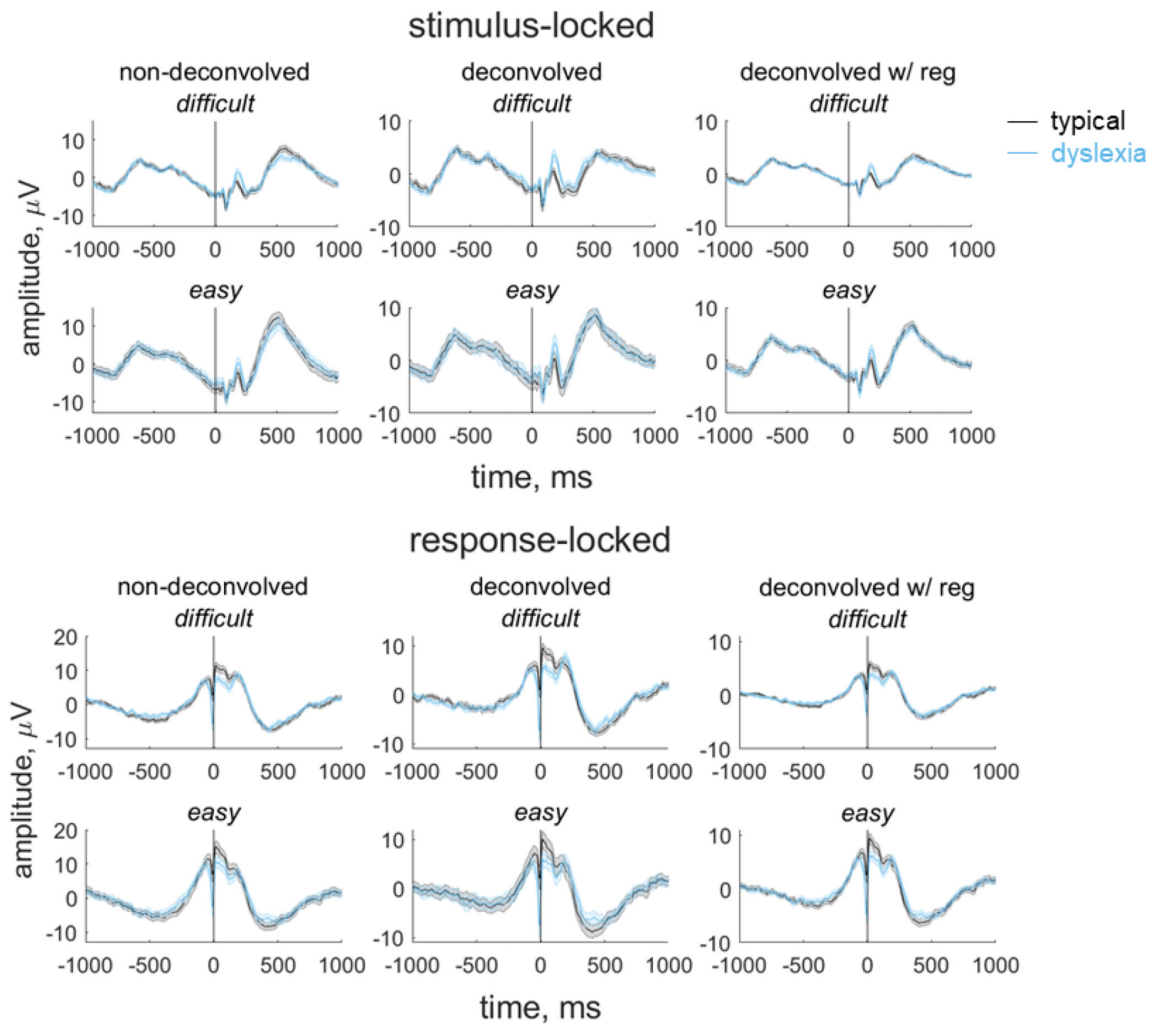
Group average stimulus-locked and response-locked evoked potentials for the motion coherence task Average (±1SEM) stimulus-locked (upper) and response-locked (lower) evoked potentials for typically developing children (grey) and children with dyslexia (blue) in the motion coherence task for difficult and easy levels. The left column shows non-deconvolved group average waveforms. The central column shows deconvolved group average waveforms (without regularisation). The right column shows deconvolved group average waveforms with regularisation (ridge regression). The vertical line at 0 ms indicates when the stimulus phase started (stimulus-locked) or when the response was made (response-locked).

**Figure 5:**
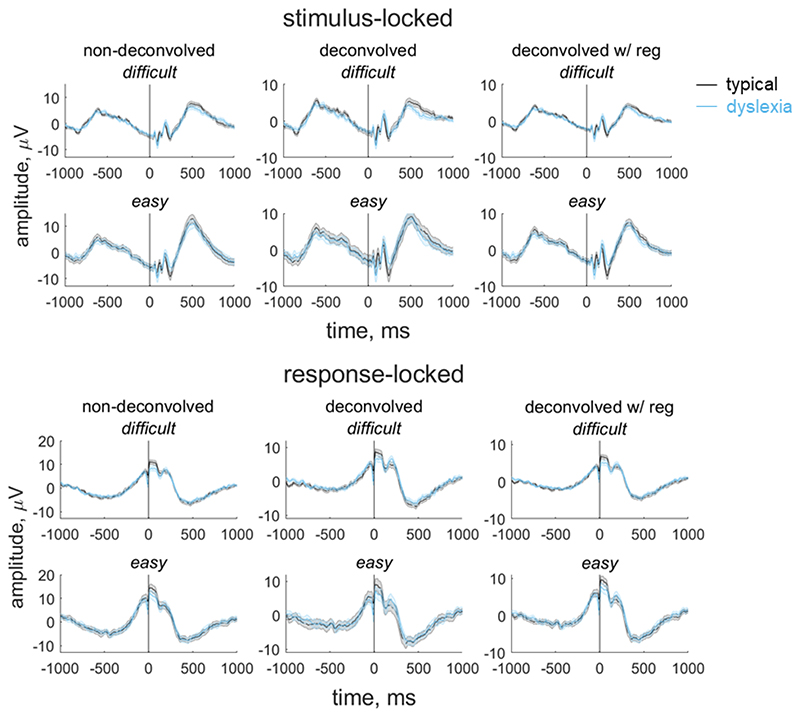
Group average stimulus-locked and response-locked evoked potentials for the direction integration task Average (±1SEM) stimulus-locked (upper) and response-locked (lower) evoked potentials for typically developing children (grey) and children with dyslexia (blue) in the direction integration task for difficult and easy levels. The left column shows non-deconvolved group average waveforms. The central column shows deconvolved group average waveforms (without regularisation). The right column shows deconvolved group average waveforms with regularisation (ridge regression). The vertical line at 0 ms indicates when the stimulus phase started (stimulus-locked) or when the response was made (response-locked).

In our paradigm, stimulus-locked and response-locked activity overlap temporally, with the degree of overlap relating to the participant’s reaction time. Importantly, the extent of overlap could vary between groups and/or conditions (Ehinger & Dimigen, 2019). Thus, in order to obtain an EEG measure for inclusion in our model that reflects the decision-making process as purely as possible, and fully separate the contributions of stimulus-locked and response-locked activity, we used a linear deconvolution method to unmix overlapping stimulus-locked and response-locked activity in our component waveform using the Unfold toolbox (Ehinger & Dimigen, 2019). We modelled the continuous waveform for each participant by selecting a time window of -1000 ms to 1000 ms around each stimulus event or response event. We specified a design matrix with predictors for each difficulty level (difficult, easy) for each event type (stimulus, response). We then time-expanded the design matrix by adding a predictor for each timepoint sampled (i.e., every 4 ms from -1000 ms to 1000 ms) for each event type. The reason for this ‘time-expansion’ is that each regressor in the resulting design matrix models the evoked response (either stimulus-locked or response-locked) at a particular point in time (Smith & Kutas, 2015; Ehinger & Dimigen, 2019); this is equivalent to the ‘finite impulse response’ approach to analysis of fMRI timeseries (Henson, Rugg and Friston, 2001). The predictors are therefore simply ‘boxcar’ functions at each point in time, rather than information relating to the stimulus display. Having constructed the design matrix, we identified segments with amplitudes above ±250 μV using a sliding 2000 ms segment in 100 ms steps, and excluded these segments from the design matrix (motion coherence task: mean 2.36% of data for each participant, range: 0 to 19.50%; direction integration task: mean 2.19% of the data for each participant, range: 0 to 21.17%). We then fit the deconvolution model resulting in regression weights (betas) for each of the 2 event types, 2 difficulty levels and 500 timepoints, which we used to construct regression waveforms (see Figures 4 and 5). Comparing the left and middle columns of Figures 4 and 5 shows that deconvolution led to reduced amplitudes (which is expected as the non-deconvolved waveform contains a mix of overlapping stimulus-locked and response-locked activity).

The non-deconvolved waveforms showed amplitude differences between difficult and easy levels (Figures 4 and 5, left column), as to be expected for an EEG measure which reflects the decision-making process. However, these differences across difficulty levels were not evident in the deconvolved waveforms (Figures 4 and 5, central column). The fact that the difference between difficulty levels changed as a result of deconvolution could suggest that the overlap between stimulus- and response-locked activity differs between difficulty levels, due to different RT distributions in each difficulty level. However, we found a difficulty level difference in the non-deconvolved waveforms even when matching the RT distributions for the easy and difficult levels, so that difficulty level differences could not be purely attributed to different RT distributions. We therefore suspected that the beta estimates may be noisy and that the deconvolution technique was overfitting the noise. Therefore, in the final step where we selected EEG measures for inclusion in the diffusion model, we re-ran the deconvolution model using a regularisation method which penalises the squared magnitude of the regression coefficients (ridge regression; see Kristensen et al., 2017) to minimise noise. Using this approach retained the difficulty level differences while minimising the noise in the waveforms (see right column of Figures 4 and 5). Specifically, we found the best regularisation parameter for each participant using cross-validation, and then took the mode across all participants and constrained the regularisation parameter to ensure that differences in regularisation did not contribute to group differences in resulting waveforms. The modal parameter value was 10 for the motion coherence task (5.5 and 10 for the typically developing children and children with dyslexia, separately) and 5 for the direction integration task (5 and 4.5 for the typically developing children and children with dyslexia, separately). We then fit a regression slope to each participant’s average deconvolved waveform for each difficulty level between -200 ms to 0 ms around the time of the response to obtain a slope measure which we entered into the diffusion model and related to drift-rate.

To assess the relationship between drift-rate and the EEG component discussed above, we used a joint modelling approach (Turner et al., 2013, 2015, 2016, Evans et al., 2018; Knowles et al., 2019). Specifically, we estimated additional hyper-parameters for the correlation between the *v*.*mean* parameter and the average of the EEG measure (slope of centro-parietal component activity between -200 ms to 0 ms before response) over difficulty levels *(EEG.mean)*, and between the *v*.*diff* parameter and the difference in the EEG measure between difficulty levels *(EEG.diff)*. Specifically, this meant that the structure of the original hierarchical model (with age partialled out) was only different for the drift-rate parameter, which was now a bivariate normal with the EEG measure:

$$\begin{array}{c} \left[v_{p1}-v_{p2},EEG_{p1}-EEG_{p2}\right]∼\\ BN\left(\left[{\text{μ}}_{v.diff}\pm \delta_{v.diff},\mu_{EEG.diff}\pm \delta_{EEG.diff}\right],\left[\sigma_{v.diff}^{2},\sigma_{v.diff}\sigma_{EEG.diff}\rho ,\sigma_{EEG.diff}\sigma_{v.diff}\rho ,\sigma_{EEG.diff}^{2}\right]\right)\\ \left[\left(v_{p1}+v_{p2}\right)/2,\left(EEG_{p1}+EEG_{p2}\right)/2\right]∼\\ BN(\left[{\text{μ}}_{\text{v}.\text{mean}}\pm \delta_{\text{v}.\text{mean}},{\text{μ}}_{EEG.mean}\pm \delta_{EEG.mean}\right],\left[\sigma_{v.mean}^{2},\sigma_{v.mean}\sigma_{EEG.mean}\rho ,\sigma_{EEG.mean}\sigma_{v.mean}\rho ,\sigma_{EEG.mean}^{2}\right])\\ {\text{μ}}_{EEG.diff}∼N(0,0.5)\\ {\text{μ}}_{EEG.\text{mean}}∼N(0,1)\\ \sigma_{EEG.diff},\sigma_{EEG.mean}∼\text{Γ}(1,1)\\ \delta_{EEG.diff},\delta_{EEG.mean}∼N(0,0.01)\\ \rho ∼U(-1,1) \end{array}$$

where ***ρ*** refers to the correlation between drift-rate and the EEG measure. Note that we again used DE-MCMC with 15 interacting chains to sample from the posterior of the joint model, though due to the greater computational burden of the model we used 3000 iterations, of which the first 1000 were discarded as burn-in and no migration algorithm was implemented. Furthermore, we estimated two different variants of this joint model: one where the correlations were constrained to be the same across groups, which would allow for the estimation of more precise posteriors due to the limited sample size, and another less constrained version were the correlations were estimated separately for each group.

## Results

### Diffusion modelling of behavioural data

**Figure 6:**
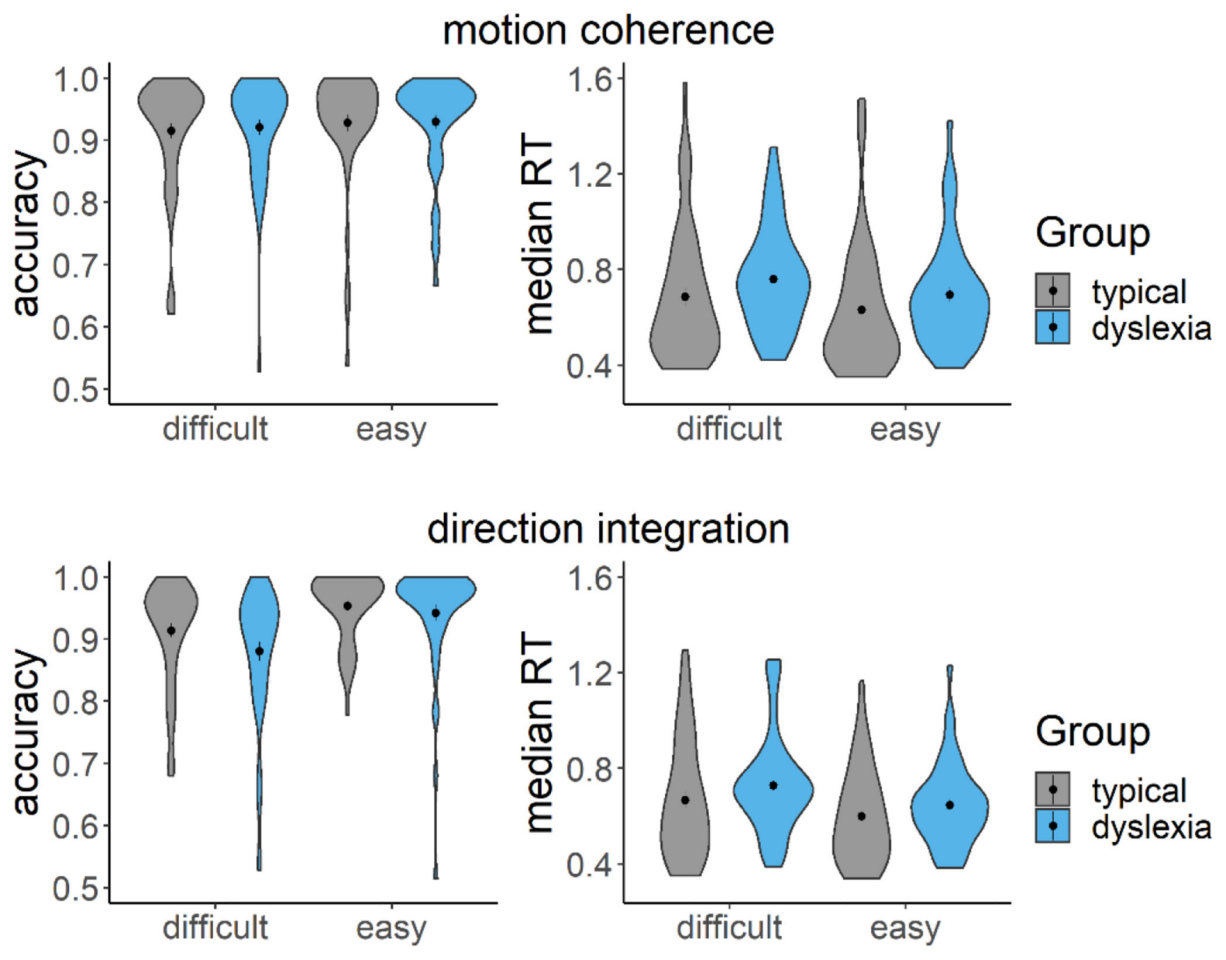
Accuracy and median response time (RT) for correct trials Violin plots showing the kernel probability density for each group’s accuracy (left) and median RT (s) for correct trials (right) for each difficulty level and each task (upper: motion coherence; lower: direction integration). Data for typically developing children and children with dyslexia are presented in grey and blue, respectively. Dots and vertical lines represent the group mean and ±1 SEM.

**Figure 7:**
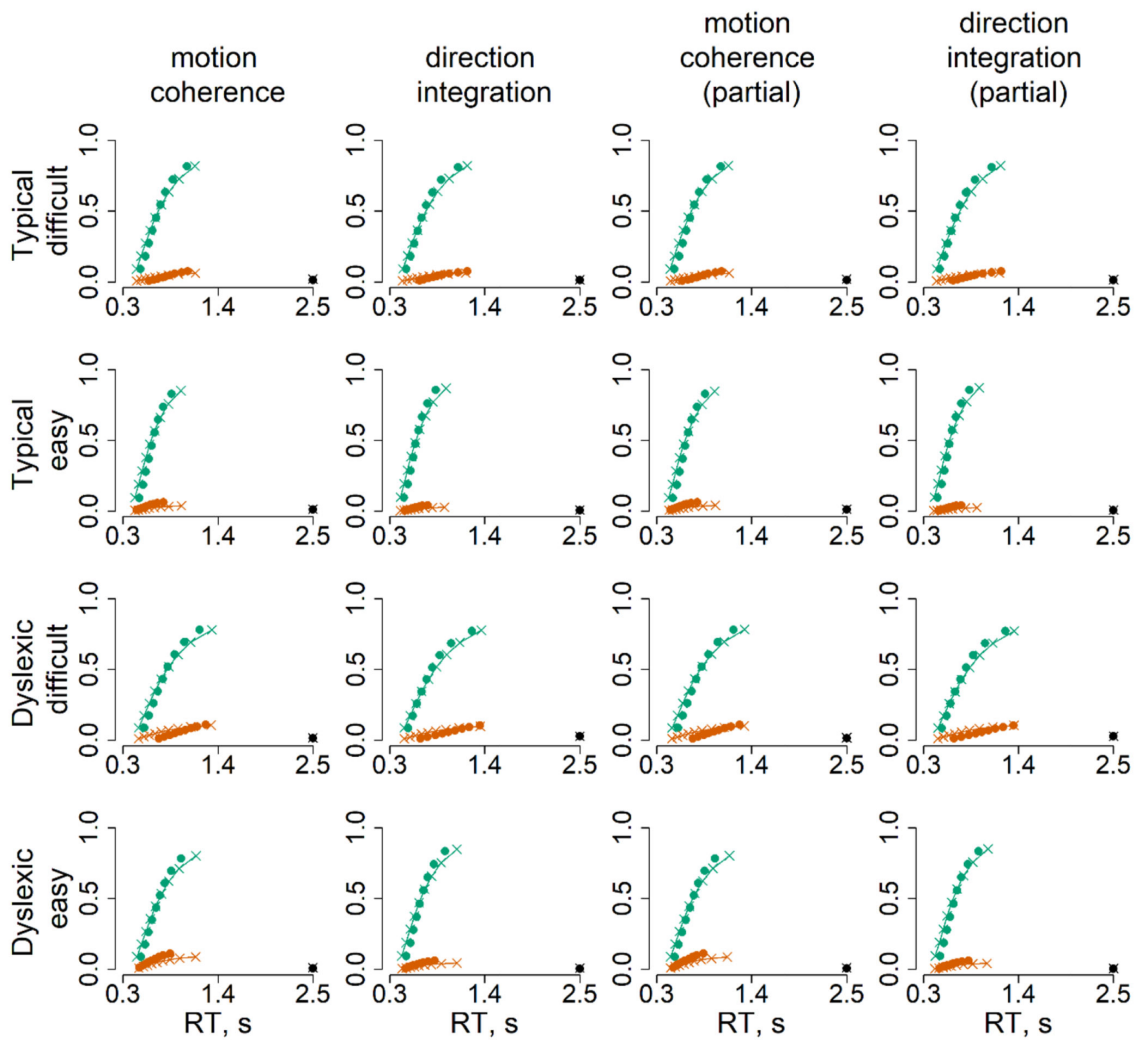
Model fits Defective cumulative density function plots for each of the four models, for typically developing children (upper rows) and children with dyslexia (bottom rows) for difficult and easy levels. Green represents correct responses and red represents error responses, at each of 9 quantiles. The dots reflect the observed data and crosses with connecting lines reflect the model fit. The dots and crosses at 2.5 seconds reflect the observed and model predicted misses.

Figure 6 summarises the accuracy and response time data subjected to diffusion modelling. This figure shows that the children with dyslexia had slightly slower median response times compared to typically developing children, on average, and were slightly less accurate in the direction integration task, particularly on the difficult trials. However, there was substantial overlap between the groups with considerable variability within each group. These behavioural data were well-fit by our diffusion models, as shown by the cumulative density functions in Figure 7. All chains were well-converged, as reflected by Gelman-Rubin diagnostic values (Gelman & Rubin, 1992) close to 1 (*M* = 1.00, range = 1.00 – 1.07).

**Figure 8:**
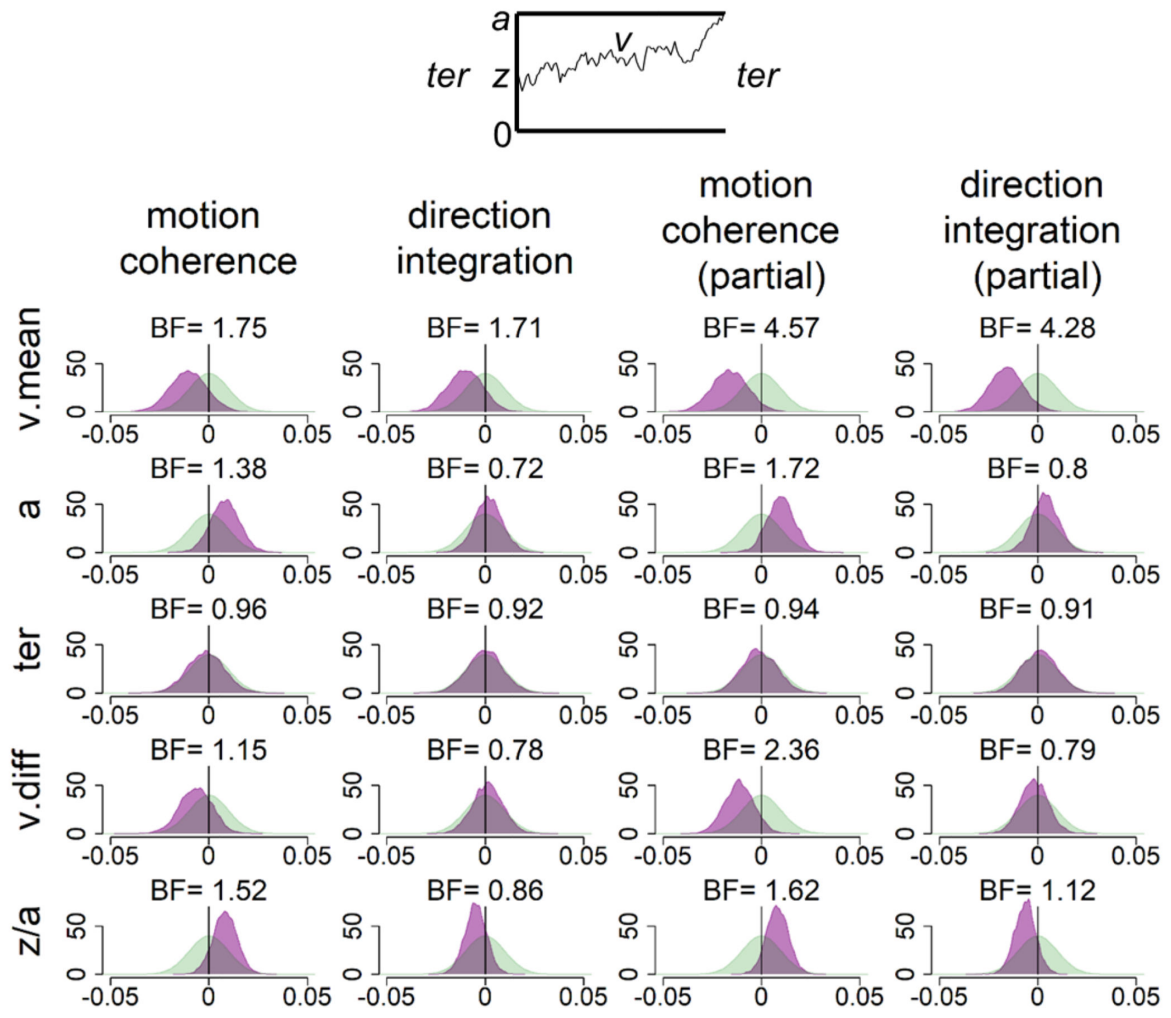
Prior and posterior density distributions Prior (green) and posterior (purple) density distributions for the group-level parameters reflecting group differences in each of the 5 model parameters (v.mean = mean drift-rate across difficulty levels; a = boundary separation; ter = non-decision time; v.diff = difference in mean drift-rate between difficulty levels; z/a = relative starting point) for each task. The upper inset shows a schematic of the model parameters shown. The leftmost columns show the results of the standard model and the rightmost columns show the results of the model with age partialled out. Negative values reflect lower parameter values in the dyslexia group compared to the typically developing group. BF = Savage-Dickey Bayes factors in favour of the alternative hypothesis (H_1_) over the null hypothesis (H_0_). BF > 1 support H_1_.

Figure 8 shows the prior and posterior distributions for the group-level parameters that reflect the difference between groups for each of the 5 parameters (*v*.*mean*, *a*, *ter*, v.diff, *beta*), along with Bayes factors. Bayes factors above 1 reflect more evidence for the alternative hypothesis of group differences compared to the null hypothesis, whereas Bayes factors below 1 reflect relatively more evidence for the null hypothesis than the alternative hypothesis. We use the heuristic that Bayes factors between 1/3 and 3 constitute only weak, inconclusive evidence (Jeffreys, 1961).

In support of our first hypothesis, children with dyslexia had reduced drift-rates in the motion coherence task compared to typically developing children, as shown by the leftward shift in the posterior distribution of *v*. *mean* in Figure 8. When age was partialled out, there was moderate evidence in favour of group differences (BF = 4.57, population effect size M = -.18, 95% CI: [-.40, .02]). The evidence was weaker when age was not partialled out (BF =1.75). Interestingly, the same pattern was found in support of our second hypothesis, with children with dyslexia also showing reduced drift-rates in the direction integration task compared to typically developing children. Again, there was moderate evidence for group differences when age was controlled for (BF = 4.28, population effect size M = -.21, 95% CI: [-.45, .02]), but weak evidence when age was not controlled for (BF = 1.71).

Our third hypothesis was that children with dyslexia would show increased boundary separation. Although children with dyslexia did have slightly higher boundary separation compared to typically developing children (indicated by a small rightward shift in the posterior distribution of *a* in Figure 8), particularly in the motion coherence task, the evidence remained inconclusive, even when controlling for age. Our final hypothesis was that there would be no group differences in non-decision time *(ter)* in either task. Figure 8 shows little difference between the groups in this parameter, but the Bayes factors are close to 1, suggesting inconclusive evidence. Therefore, more data would be required to make firm conclusions regarding these hypotheses.

**Figure 9:**
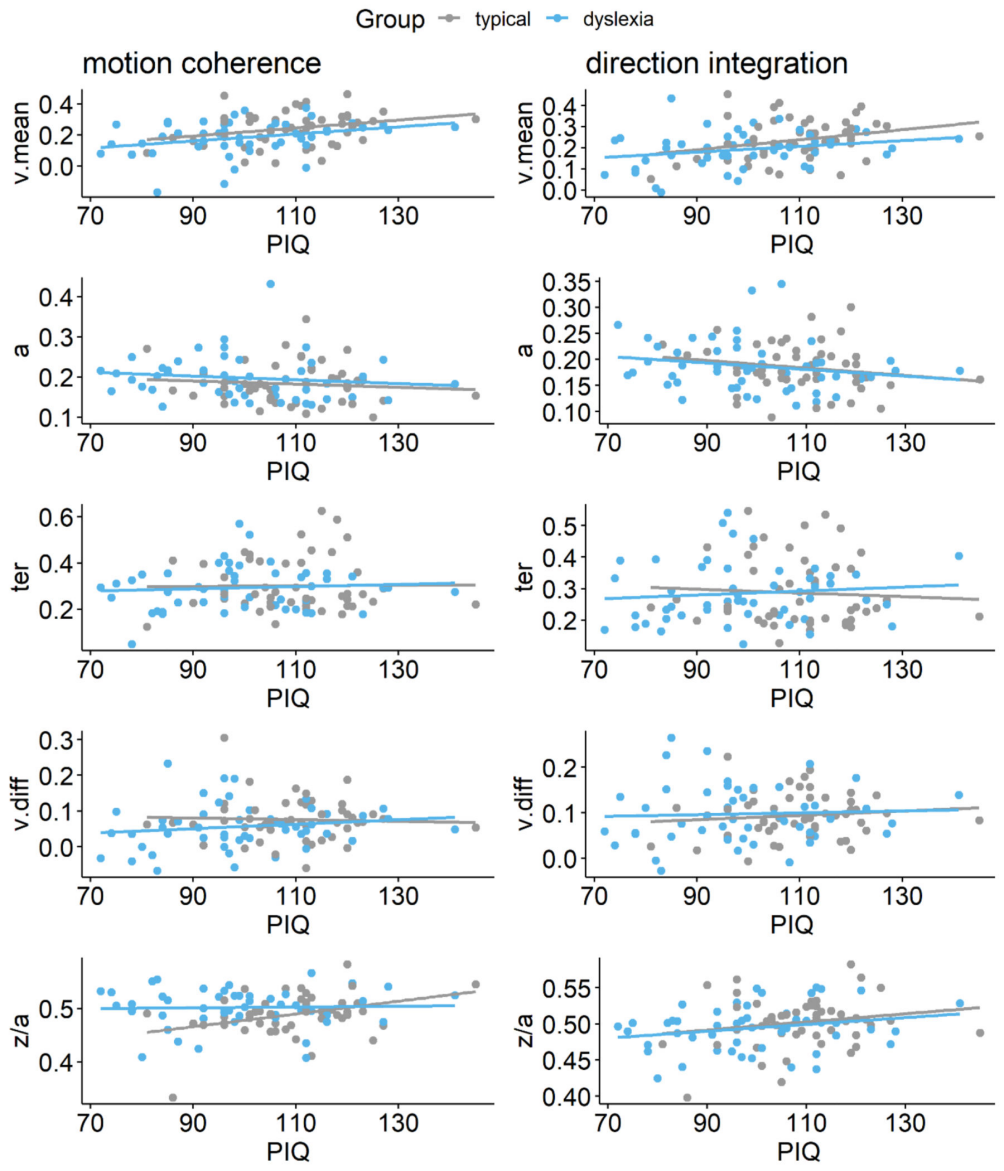
Scatterplots plotting individual parameter estimates against performance IQ Maximum likelihood estimates contained within the posterior for each participant’s mean drift-rate across difficulty levels (*v*.*mean*), boundary separation (*a)*, non-decision time (*ter*), difference in drift-rate between difficulty levels (*v*.*diff*), and starting point *(z/a)*, plotted as a function of performance IQ (PIQ), for the motion coherence task (left column) and direction integration task (right column). Typically developing children are plotted in grey and children with dyslexia are plotted in blue.

**Figure 10:**
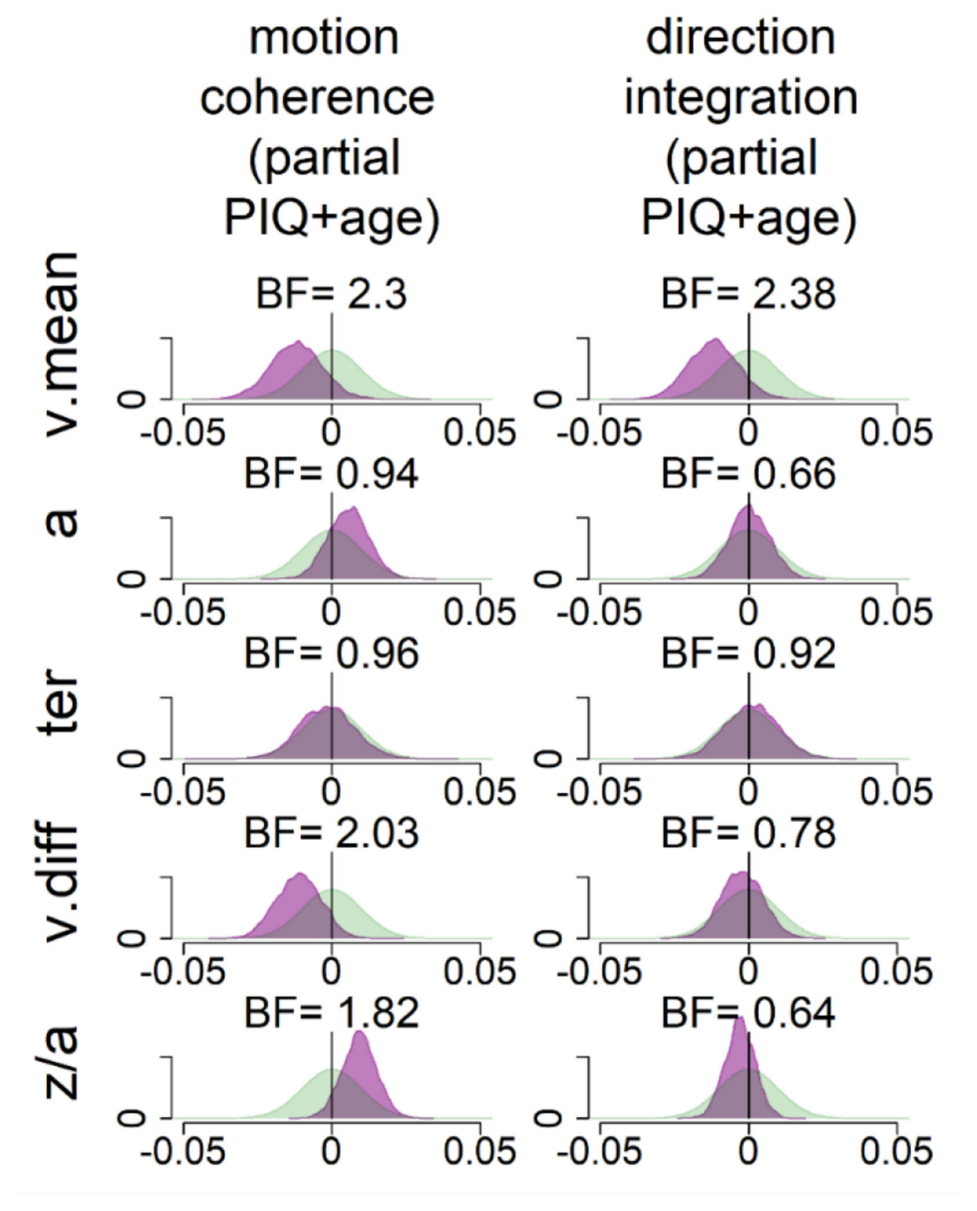
Exploratory analyses: prior and posterior density distributions for model with age and performance IQ partialled out While our pre-registered analysis did not control for performance IQ, we conducted an exploratory analysis to investigate whether group differences in drift-rate were still apparent when controlling for performance IQ. The figure shows prior (green) and posterior (purple) density distributions for the group-level parameters reflecting group differences in each of the 5 model parameters (v.mean = mean drift-rate across difficulty levels; a = boundary separation; ter = non-decision time; v.diff = difference in mean drift-rate between difficulty levels; z/a = relative starting point) for each task, when both age, performance IQ (PIQ) and their interaction are partialled out. Negative values reflect lower parameter values in the dyslexia group compared to the typically developing group. BF = Savage-Dickey Bayes factors in favour of the alternative hypothesis (H_1_) over the null hypothesis (H_0_). BF > 1 support H_1_. As in Figure 8, the posterior distribution for *v*.*mean* is shifted leftwards, reflecting lower mean drift-rate in the dyslexia group than the typically developing group. The corresponding Bayes factors are smaller in these analyses, indicating weaker evidence for group differences. As we reflect on in the Discussion of the main manuscript, the decision to partial out PIQ should not be taken lightly, as PIQ seems to contribute to both decision making variables (drift-rate) and group differences, so it is likely that partialling out PIQ removes some of the variance related to the group differences we are interested in.

These pre-registered analyses did not control for performance IQ because it could be meaningfully related to both decision-making parameters and group membership, and investigating its contribution to both was beyond the scope of our multi-level modelling approach. However, as there was an indication of a relationship between performance IQ and drift-rate (Figure 9), and as both performance IQ and drift-rate differed between the groups, we investigated these links further with an exploratory analysis which partialled out the effects of both age and performance IQ (Figure 10). In brief, BFs of 2.3 and 2.38 in the two tasks continue to provide weak evidence for group differences in mean drift-rate when both age and PIQ are controlled for.

### Joint modelling of EEG and behavioural data

**Figure 11:**
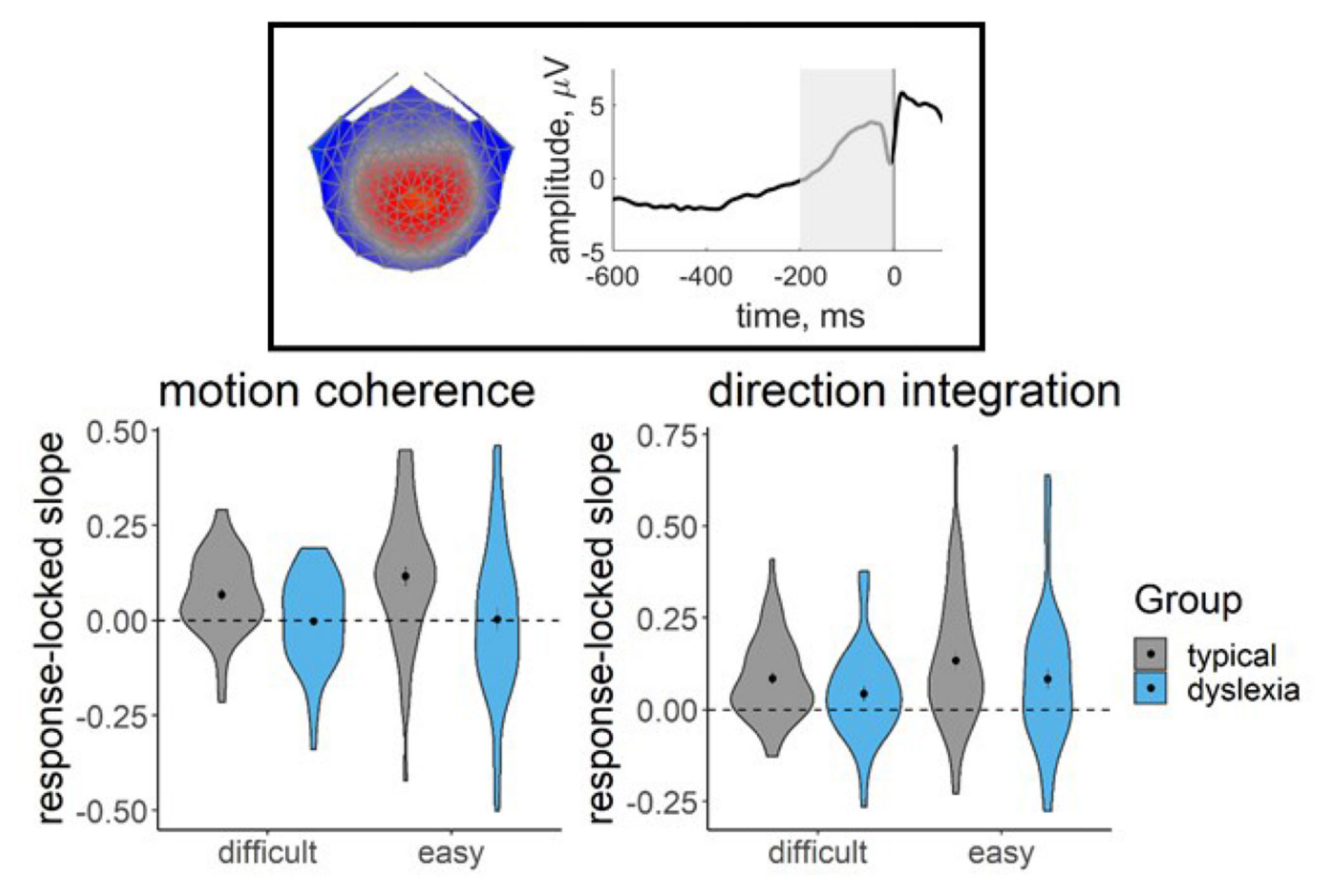
EEG slope measure extracted for inclusion in the joint model Violin plots showing the kernel probability density for the EEG slope measure extracted for inclusion in the joint model for each group (typically developing: grey; dyslexia: blue) for each difficulty level. The extracted measure was the slope of a linear regression line fitted to each participant’s deconvolved (with regularisation) response-locked waveform, from 200 ms prior to the response to the response (see shaded area of schematic response-locked waveform in inset). The dotted line reflects a flat slope. Dots and vertical lines represent the group mean and ±1 SEM.

Figure 11 shows the distribution of slope measures that were extracted from each participant’s deconvolved (with regularisation) response-locked waveform, which were used in joint modelling to explore links between EEG and model parameters. While there was considerable between-participants variability, the children with dyslexia had shallower slopes than the typical children, on average. A Bayesian repeated measures ANOVA in JASP (JASP Team, 2020) showed that, in the motion coherence task, the best model of EEG slope measures included both the within-participants factor of difficulty level, the between-participants factor of group and an interaction term. When averaging across models, there was strong evidence for including a main effect of group (BF_incl_ = 14.70) and a group by difficulty level interaction (BF_incl_ = 4.65). Yet in the direction integration task, the best model of EEG slope measures included only the within-participants factor of difficulty, with inconclusive evidence for including a main effect of group (BF_incl_ = 0.70) or a group by difficulty level interaction (BF_incl_ = 0.49). Therefore it seems that the build-up of activity in the centro-parietal component is clearly reduced in children with dyslexia in the motion coherence task, but the reduction is not compelling in the direction integration task.

**Figure 12:**
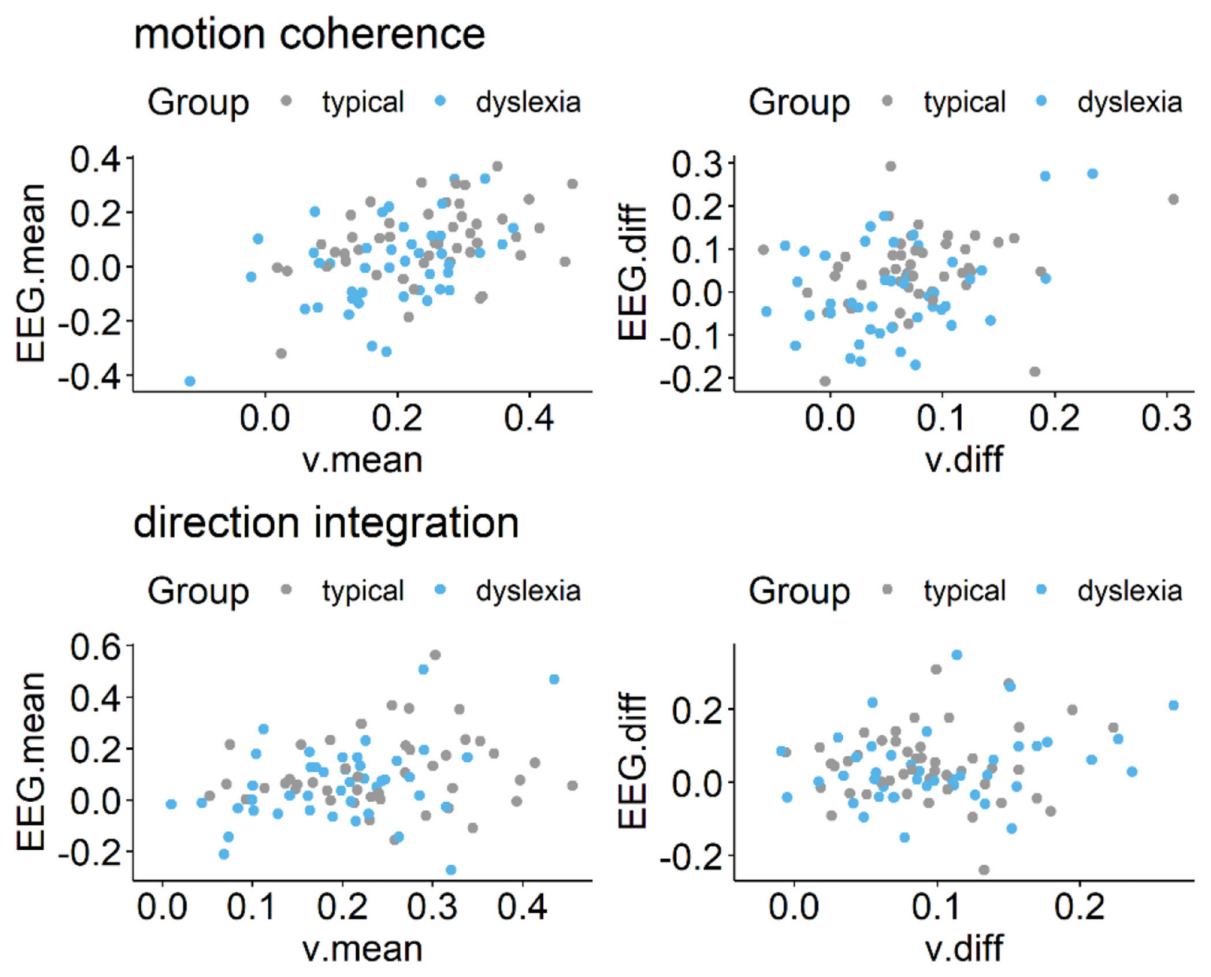
Scatterplots showing relationship between drift-rate and EEG Left panels show maximum likelihood estimates contained within the posterior for each participant’s mean drift-rate across difficulty levels (*v*.*mean*) plotted against the slope of EEG activity averaged across difficulty levels (*EEG*.*mean*) for the motion coherence (top) and direction integration (bottom) tasks. Right panels show point estimates for each participant’s difference in drift-rate between difficulty levels (*v*.*diff*) plotted against the difference in slopes of EEG activity between the two difficulty levels (*EEG*.*diff*), for each task. Typically developing children are plotted in grey and children with dyslexia are plotted in blue.

Next we established whether this EEG measure was related to drift-rate across the whole sample, estimating a single correlation for both groups, with the effects of age partialled out. For both tasks, the EEG measure was positively related to both the mean drift-rate across difficulty levels, though the evidence was only weak in the case of the direction integration task (motion coherence: posterior mean *r* = .44, 95% credible intervals (CI) = [.26, .6], BF = 8869.49; direction integration: posterior mean *r* = .25, CI = [.03, .45], BF = 1.65). The posterior means were in the direction of a positive relationship between the difference in EEG measure and the difference in drift rate between difficulty levels, although the evidence was inconclusive with relatively more evidence for the null hypothesis (motion coherence: posterior mean *r* = .22, CI = [-.02, .44], BF = .73; direction integration: posterior mean *r* = .17, CI = [-.08, .4], BF = 0.43; see Figure 12 for scatterplots).

**Figure 13:**
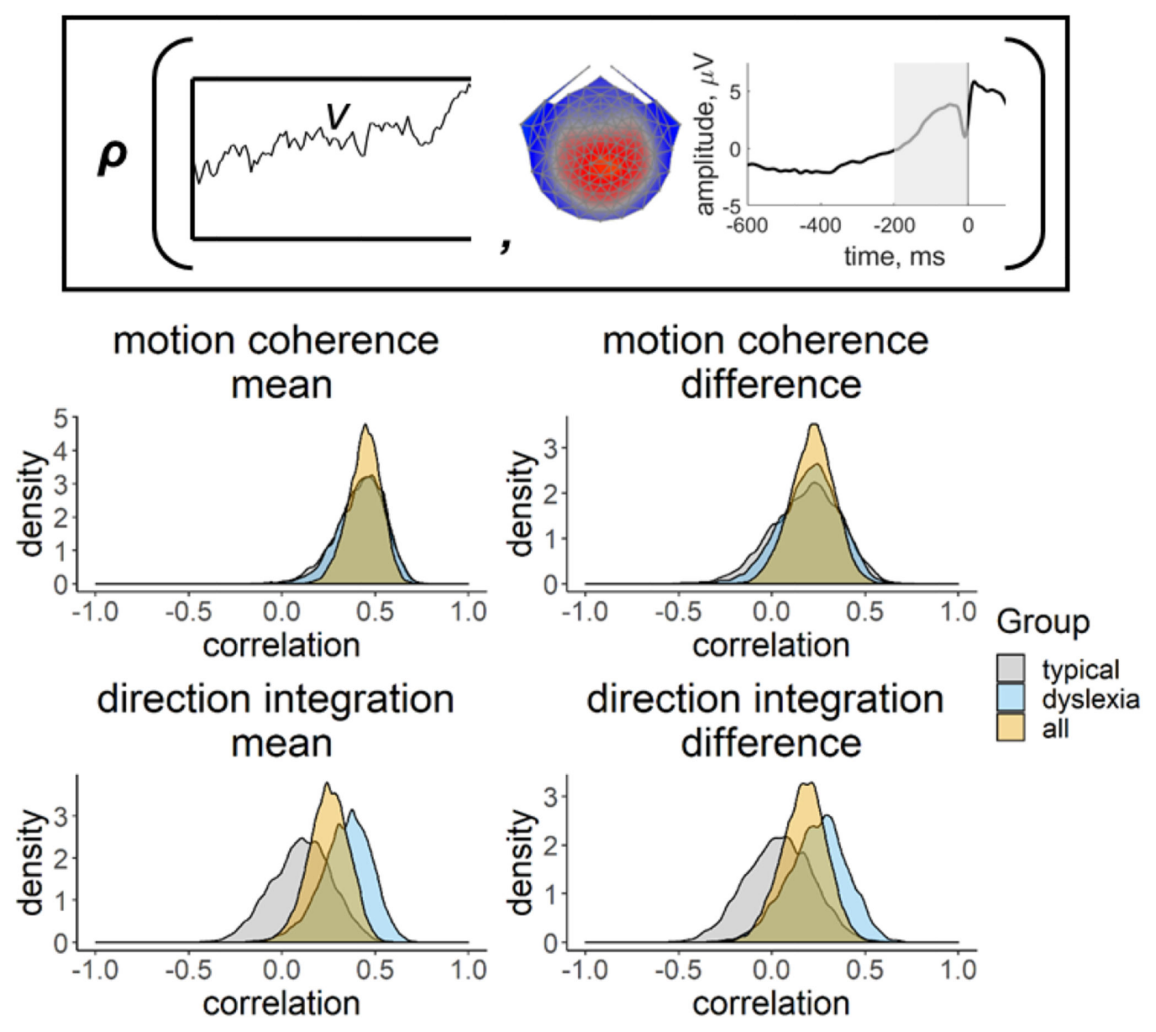
Posterior density plots showing the correlation between drift-rate and the EEG measure Inset provides a schematic representation of the drift-rate parameter (*v*; left) and EEG measure (slope of response-locked waveform from -200 ms to 0 ms around the response; right) that were correlated in the joint model, where ***ρ*** represents the correlation. Posterior density plots in the left column reflect the correlation between the mean drift-rate across difficulty levels *(v.mean)* and the mean EEG slope measure across difficulty levels (*EEG*.*mean*). Posterior density plots in the right column reflect the correlation between the difference in drift-rate between difficulty levels (*v*.*diff*) and the difference in EEG slope measure between difficulty levels (*EEG*.*diff*). Plots for the motion coherence task are presented in the upper row and plots for the direction integration task are presented in the lower row. The orange distribution shows the correlation across all participants, and the grey and blue distributions show separate correlations estimated for typical children and children with dyslexia, respectively.

Next we fit joint models in which we estimated a separate correlation coefficient between drift-rate and the EEG measure for the children with dyslexia and typical children (Figure 13). Note that our intention was not to explicitly test for differences in correlations between groups, but rather to see if the previous findings seem to hold for each group; any separation between the groups below is intended to merely describe our estimated posterior distributions. A positive correlation can be seen for both groups in the motion coherence task for the mean drift-rate across difficulty levels (typical: posterior mean *r* = .41, CI = [.13, .63], BF = 7.45; dyslexia: posterior mean *r* = .43, CI = [.15, .64], BF = 12.75). The posterior means were in the direction of a positive relationship for the difference in drift-rate between difficulty levels, but the evidence was inconclusive with relatively more evidence for the null hypothesis (typical: posterior mean *r* = .18, CI = [-.2, .51], BF = .39; dyslexia: posterior mean *r* = .20, CI = [-.12, .49], BF = .46). The strength of correlations was weaker in the direction integration task, particularly for the typical children, for whom the Bayes factors suggested moderate evidence for no relationship (mean drift-rate across difficulty levels: posterior mean *r* = .10, CI = [-.22, .4], BF = .29; difference between difficulty levels: posterior mean *r* = .04, CI = [-.31, .38], BF = .24). The strength of the correlations in children with dyslexia were slightly stronger than in the typical children, with the mean drift-rate across difficulty levels showing weak evidence for a relationship, though the difference in drift-rate between difficulty levels showed weak evidence for no relationship (mean drift-rate across difficulty levels: posterior mean *r* = .34, CI = [.04, .58], BF = 2.59; difference between difficulty levels: posterior mean *r* = .24, CI = [-.09, .53], BF = .61).

## Discussion

We analysed the performance of children with dyslexia and typical children in two global motion tasks using diffusion modelling, to identify the processing stages that are altered in dyslexia. In both the motion coherence and direction integration tasks, children with dyslexia accumulated sensory evidence more slowly than typical children, on average, once controlling for age. Moreover, we found a neural correlate of this evidence accumulation process that was attenuated in dyslexia in the motion coherence task, thus linking brain and behavioural measures with a latent model parameter.

The finding of reduced evidence accumulation for children with dyslexia during the motion coherence task echoes O’Brien and Yeatman (2020) and helps to explain previous reports of elevated motion coherence thresholds in dyslexia (Benassi et al., 2010). Importantly, the current study goes further by showing that reduced evidence accumulation is also found in a direction integration task that does not require segregating signal dots from noise dots. This result suggests that dyslexic individuals have general difficulties with extracting global motion information, rather than solely difficulties with noise exclusion (cf. Conlon et al., 2012; Sperling et al., 2006) –in line with reports of atypical performance in an illusory motion task without noise exclusion requirements (Gori et al., 2015, 2016). These general difficulties could reflect reduced temporal and/or spatial integration of motion signals (Benassi et al., 2010; Hill & Raymond, 2002; Raymond & Sorensen, 1998). This conclusion does not negate the possibility that dyslexic individuals face additional difficulties when segregating signal from noise, as we suggested based on stimulus-locked analyses using a similar dataset (Toffoli et al., 2021).

By supplementing our diffusion modelling analysis with EEG, we identified a neural index of reduced evidence accumulation in dyslexia. Specifically, we used a data-driven component decomposition technique to find a centro-parietal component previously linked to decision-making (Kelly and O’Connell, 2013; O’Connell et al., 2012; Manning et al., 2021a), and then ‘unmixed’ overlapping stimulus- and response-locked activity. In the motion coherence task, we found that children with dyslexia showed a shallower build-up in the response-locked centro-parietal component compared to typical children, and the gradient of the build-up was positively correlated with drift-rate in the joint model. While the EEG analysis was exploratory, the results are consistent with an earlier study of typically developing children (Manning et al., 2021a) and follow our hypothesised pattern (https://osf.io/enkwm). Similarly, Stefanac et al. (2021) reported reduced centro-parietal build-up in children with dyslexia compared to chronological and reading age-matched controls. Yet, in our direction integration task, we found no compelling evidence for reduced centro-parietal build-up in children with dyslexia and the evidence for a relationship between this EEG measure and drift-rate was weaker. This suggests that the magnitude of the centro-parietal positivity and its association with drift-rate may be group- and task-dependent, to some extent (see also Lui et al., 2021).

Alongside reductions in drift-rate, we hypothesised that children with dyslexia would show wider boundary separation compared to typically developing children, reflecting more cautious responses, and no differences in non-decision time. We found some evidence for increased boundary separation in children with dyslexia in the motion coherence task, but this was inconclusive. There was also inconclusive evidence for group differences in non-decision time. These results are not at odds with O’Brien and Yeatman (2020), but suggest that more data are required to reach a firm conclusion regarding these parameters. Seemingly any group differences in these parameters are more subtle than group differences in drift-rate. We note that the inferential method used by O’Brien and Yeatman (2020) differed from our own: while they also fit a hierarchical Bayesian model, they then extracted point estimates of diffusion model parameters for each individual to draw statistical inferences. Importantly, this means that O’Brien and Yeatman (2020) ignored the uncertainty in the individual-level parameters, which can inflate the evidence in favour of the winning model (Boehm et al., 2018; Evans & Wagenmakers, 2019).

Together with the results from stimulus-locked analyses using a similar dataset (Toffoli et al., 2021), our results suggest that early sensory encoding of motion information is not altered in children with dyslexia. While differences in drift-rate cannot completely tease apart sensory and decision-making processes, in the current study we found no evidence of group differences in non-decision time – a measure which includes the time taken for sensory encoding. Moreover, Toffoli et al. showed that early peaks reflecting motion-specific processing were similar in children with dyslexia and typically developing children, with differences arising only after ~430 ms following stimulus onset, specifically in the motion coherence task. The current analyses suggest that differences in dyslexia arise due to the efficiency with which evidence is extracted from global motion stimuli and integrated towards a decision bound, which is often attributed to parietal areas (Hanks et al., 2006; Shadlen & Newsome, 1996; 2001; de Lafuente et al., 2015). Without a comparable form task, it is unclear from the current study whether reduced evidence accumulation is restricted to tasks that tax the dorsal stream. However, we suggest that *within* the magnocellular/dorsal stream, early sensory processing is unaffected in dyslexia with group differences emerging only at later processing stages, including those involved in decision-making. While this conclusion contrasts studies indicating early alterations of the magnocellular pathway in dyslexia (Giraldo-Chica et al., 2015; Livingstone et al., 1991; Perani et al., 2021; Stein, 2001, 2019; Stein & Walsh, 1997), the global motion tasks used in the current study are not ideally placed to isolate magnocellular processes (Skottun, 2011; Skottun & Skoyles, 2006, 2008; Skottun, 2016). Future work will be required to determine how specific reduced evidence accumulation in dyslexia is to visual motion processing. Slower responses have been reported in dyslexia for other tasks (Catts et al., 2002, Nicolson & Fawcett, 1994) which could reflect pervasive reduced evidence accumulation, and reduced global integrative processes have been reported in static tasks in children with dyslexia (Franceschini et al., 2017a). However, slowed responses could arise for different reasons (e.g., increased non-decision time, wider boundary separation), so diffusion model decompositions on various tasks are required.

A number of future research directions emerge. What cognitive skills other than magnocellular / dorsal stream processing contribute to reduced drift-rate in dyslexia? General processing speed is a unique predictor of word reading and comprehension (Christopher et al., 2012) and RAN is a recognized independent contributor to variation in reading ability, complementing phonological skills (e.g., O’Brien & Yeatman, 2020). Future work will need to establish the extent to which reduced processing speed and slower RAN associate with reduced drift-rate in dyslexia. Additionally, performance IQ varied across our two groups and was associated with drift-rate. Exploratory models revealed that, even when controlling for both age and performance IQ, there was still relatively more evidence for group differences in drift-rate than no group differences. Yet the evidence was weaker than in models controlling only for age. Importantly, partialling out differences in performance IQ could remove some of the variance related to the group differences we are interested in, as atypical development could lead to both dyslexia and reduced IQ (Dennis et al., 2009). Indeed, performance IQ has been shown to strongly predict reading skills, independently of phonological skills (O’Brien & Yeatman, 2020). Future work will need to investigate the contribution of processing speed and performance IQ to decision making across the spectrum of reading abilities. Future research will also be required to explain the considerable between-participants variability in model and EEG parameters in children with and without dyslexia.

By combining diffusion modelling and EEG measures that are sensitive to the multiple processes contributing to motion perception, we have uncovered differences between children with dyslexia and typically developing children that could not be observed in behavioural responses alone. Moreover, diffusion modelling allows motion sensitivity to be measured without confounding speed-accuracy tradeoffs. Given that reduced behavioural sensitivity to motion has been reported in a range of other disorders (Braddick et al., 2003; Chen et al., 2003; McKendrick & Badcock, 2004), we suggest that diffusion modelling may provide a useful framework to identify convergence and divergence across different conditions, with implications for understanding the development of these conditions and their relationship to other cognitive processes.

Future work should establish whether differences in evidence accumulation of motion information contribute causally to the reading difficulties experienced by children with dyslexia. Some studies have suggested a causal relationship between motion perception and reading difficulties (e.g., Boets et al., 2011; Ebrahimi et al., 2019; Gori et al., 2016; Kevan & Pammer, 2009; Lawton, 2016; Qian & Bi, 2015), so it would be interesting to know if evidence accumulation processes can be trained to improve reading ability. In support of this possibility, action video game training has been shown to improve motion perception by acting on the evidence accumulation phase (Green et al., 2010) and action video game training has also been linked to improved reading skills in children with dyslexia (Franceschini et al., 2013; 2017b, Franceschini & Bertoni, 2019; Bertoni et al., 2019; 2021). Such causal links will need to be investigated in future work using training or intervention designs.

## Figures and Tables

**Table 1:** Demographics of participants included in final dataset

	Typically developing (n = 50)	Dyslexia (n = 50)
Age	10.65 (2.34) 6.55 – 14.98	11.08 (1.87) 7.81 – 14.53
Performance IQ	109.26 (11.53) 81 – 145	99.40 (15.29) 72 – 141
Verbal IQ	110.60 (8.42) 95 – 127	98.56 (10.60) 77 – 118
Full-scale IQ	111.36 (9.02) 89 – 132	98.70 (12.85) 75 – 132
TOWRE-2 PDE	111.18 (16.53) 81 – 153	79.16 (9.45) 51 – 99
WIAT-Spelling	105.74 (10.21) 80 – 127	77.86 (7.96) 58 – 99
Composite score	108.46 (12.15) 89.5 – 138.0	78.51 (7.46) 54.5 – 89.0

Note. Data are presented as M (SD) Range.

## Data Availability

Analysis scripts and output files are available at: https://osf.io/nvwf7/. Data and output files are available on the UK Data Service at http://doi.org/10.5255/UKDA-SN-855236.

## Significance statement

Reduced sensitivity to visual information has been reported in dyslexia, with a lively debate about whether these differences causally contribute to reading difficulties. In this large pre-registered study with a blind modelling approach, we combine state-of-the art methods in both computational modelling and EEG analysis to pinpoint the stages of processing that are atypical in children with dyslexia in two visual motion tasks that vary in their requirement for noise exclusion. We find reduced evidence accumulation in children with dyslexia across both tasks, and identify a neural marker, allowing us to link brain and behaviour. We show that children with dyslexia exhibit general difficulties with extracting sensory evidence from global motion displays, not just in tasks that require noise exclusion.

## References

[R1] Benassi M, Simonelli L, Giovagnoli S, Bolzani R (2010). Coherence motion perception in developmental dyslexia: A meta-analysis of behavioural studies. Dyslexia.

[R2] Bertoni S, Franceschini S, Puccio G, Mancarella M, Gori S, Facoetti A (2021). Action video games enhance attentional control and phonological decoding in children with developmental dyslexia. Brain Sciences.

[R3] Bertoni S, Franceschini S, Ronconi L, Gori S, Facoetti A (2019). Is excessive visual crowding causally linked to developmental dyslexia?. Neuropsychologia.

[R4] Boehm U, Marsman M, Matzke D, Wagenmakers E-J (2018). On the importance of avoiding shortcuts in applying cognitive models to hierarchical data. Behavior Research Methods.

[R5] Boets B, Vandermosten M, Cornelissen P, Wouters J, Ghesquière P (2011). Coherent motion sensitivity and reading development in the transition from prereading to reading stage. Child development.

[R6] Bonifacci P, Snowling MJ (2008). Speed of processing and reading disability: A cross-linguistic investigation of dyslexia and borderline intellectual functioning. Cognition.

[R7] Braddick O, Atkinson J, Wattam-Bell J (2003). Normal and anomalous development of visual motion processing: motion coherence and ‘dorsal-stream vulnerability’. Neuropsychologia.

[R8] Catts HW, Gillispie M, Leonard LB, Kail RV, Miller CA (2002). The role of speed of processing, rapid naming, and phonological awareness in reading achievement. Journal of Learning Disabilities.

[R9] Chen Y, Nakayama K, Levy D, Matthysse S, Holzman P (2003). Processing of global, but not local, motion direction is deficient in schizophrenia. Schizophrenia Research.

[R10] Christopher ME, Miyake A, Keenan JM, Pennington B, DeFries JC, Wadsworth SJ, Olson RK (2012). Predicting word reading and comprehension with executive function and speed measures across development: a latent variable analysis. Journal of Experimental Psychology: General.

[R11] Conlon EG, Lilleskaret G, Wright CM, Power GF (2012). The influence of contrast on coherent motion processing in dyslexia. Neuropsychologia.

[R12] de Lafuente V, Jazayeri M, Shadlen MN (2015). Representation of accumulating evidence for a decision in two parietal areas. Journal of Neuroscience.

[R13] Dennis M, Francis DJ, Cirino PT, Schachar R, Barnes MA, Fletcher JM (2009). Why IQ is not a covariate in cognitive studies of neurodevelopmental disorders. Journal of the International Neuropsychological Society.

[R14] Dmochowski JP, Norcia AM (2015). Cortical components of reaction-time during perceptual decisions in humans. PloS one.

[R15] Dutilh G, Vandekerckhove J, Ly A, Matzke D, Pedroni A, Frey R (2017). A test of the diffusion model explanation for the worst performance rule using preregistration and blinding. Attention, Perception, & Psychophysics.

[R16] Ebrahimi L, Pouretemad H, Khatibi A, Stein J (2019). Magnocellular based visual motion training improves reading in Persian. Scientific reports.

[R17] Edwards AA, Schatschneider C (2020). Magnocellular Pathway and Reading Rate: An Equivalence Test Analysis. Scientific Studies of Reading.

[R18] Ehinger BV, Dimigen O (2019). Unfold: an integrated toolbox for overlap correction, non-linear modelling, and regression-based EEG analysis. PeerJ.

[R19] Evans NJ (2019). Assessing the practical differences between model selection methods in inferences about choice response time tasks. Psychonomic Bulletin & Review.

[R20] Evans NJ, Bennett AJ, Brown SD (2019). Optimal or not; depends on the task. Psychonomic Bulletin & Review.

[R21] Evans NJ, Brown SD (2017). People adopt optimal policies in simple decision-making, after practice and guidance. Psychonomic Bulletin & Review.

[R22] Evans NJ, Hawkins GE (2019). When humans behave like monkeys: Feedback delays and extensive practice increase the efficiency of speeded decisions. Cognition.

[R23] Evans NJ, Steyvers M, Brown SD (2018). Modeling the covariance structure of complex datasets using cognitive models: An application to individual differences and the heritability of cognitive ability. Cognitive Science.

[R24] Evans NJ, Wagenmakers E-J (2019). Theoretically meaningful models can answer clinically relevant questions. Brain.

[R25] Evans NJ, Wagenmakers E-J (2020). Evidence accumulation models: Current limitations and future directions. The Quantitative Methods for Psychology.

[R26] Franceschini S, Bertoni S (2019). Improving action video games abilities increases the phonological decoding speed and phonological short-term memory in children with developmental dyslexia. Neuropsychologia.

[R27] Franceschini S, Bertoni S, Gianesini T, Gori S, Facoetti A (2017a). A different vision of dyslexia: Local precedence on global perception. Scientific Reports.

[R28] Franceschini S, Gori S, Ruffino M, Viola S, Molteni M, Facoetti A (2013). Action video games make dyslexic children read better. Current Biology.

[R29] Franceschini S, Trevisan P, Ronconi L, Bertoni S, Colmar S, Double K, Facoetti A, Gori S (2017b). Action video games improve reading abilities and visual-to-auditory attentional shifting in English-speaking children with dyslexia. Scientific Reports.

[R30] Gelman A, Rubin DB (1992). Inference from iterative simulation using multiple sequences. Statistical Science.

[R31] Giraldo-Chica M, Hegarty JP, Schneider KA (2015). Morphological differences in the lateral geniculate nucleus associated with dyslexia. NeuroImage: Clinical.

[R32] Gori S, Mascheretti S, Giora E, Ronconi L, Ruffino M, Quadrelli E, Facoetti A, Marino C (2015). The DCDC2 Intron 2 deletion impairs illusory motion perception unveiling the selective role of magnocellular-dorsal Stream in reading (dis)ability. Cerebral Cortex.

[R33] Gori S, Seitz AR, Ronconi L, Franceschini S, Facoetti A (2016). Multiple causal links between magnocellular-dorsal pathway deficit and developmental dyslexia. Cerebral Cortex.

[R34] Goswami U (2015). Sensory theories of developmental dyslexia: three challenges for research. Nature Reviews Neuroscience.

[R35] Green CS, Pouget A, Bavelier D (2010). Improved probabilistic inference as a general learning mechanism with action video games. Current Biology.

[R36] Hanks TD, Ditterich J, Shadlen MN (2006). Microstimulation of macaque area LIP affects decision-making in a motion discrimination task. Nature Neuroscience.

[R37] Hansen PC, Stein JF, Orde SR, Winter JL, Talcott JB (2001). Are dyslexics’ visual deficits limited to measures of dorsal stream function?. NeuroReport.

[R38] Henson R, Rugg MD, Friston KJ (2001). The choice of basis functions in event-related fMRI. NeuroImage.

[R39] Hill GT, Raymond JE (2002). Deficits of motion transparency perception in adult developmental dyslexics with normal unidirectional motion sensitivity. Vision Research.

[R40] Hinshelwood J (1896). A case of dyslexia: a peculiar form of word-blindness. 1. The Lancet.

[R41] Ho DE, Imai K, King G, Stuart EA (2011). MatchIt: Nonparametric preprocessing for parametric causal inference. Journal of Statistical Software.

[R42] Howard ZL, Evans NJ, Innes RJ, Brown SD, Eidels A (2020). How is multi-tasking different from increased difficulty?. Psychonomic Bulletin & Review.

[R43] JASP Team (2020). JASP (Version 0141).

[R44] Jeffreys H (1961). Theory of probability.

[R45] Johnston R, Pitchford NJ, Roach NW, Ledgeway T (2016). Why is the processing of global motion impaired in adults with developmental dyslexia?. Brain and Cognition.

[R46] Joo SJ, Donnelly PM, Yeatman JD (2017). The causal relationship between dyslexia and motion perception reconsidered. Scientific reports.

[R47] Kelly SP, O’Connell RG (2013). Internal and external influences on the rate of sensory evidence accumulation in the human brain. Journal of Neuroscience.

[R48] Kevan A, Pammer K (2009). Predicting early reading skills from pre-reading measures of dorsal stream functioning. Neuropsychologia.

[R49] Knowles JP, Evans NJ, Burke D (2019). Some evidence for an association between early life adversity and decision urgency. Frontiers in Psychology.

[R50] Kristensen E, Guerin-Dugué A, Rivet B (2017). Regularization and a general linear model for event-related potential estimation. Behavior Research Methods.

[R51] Lawton T (2016). Improving dorsal stream function in dyslexics by training figure/ground motion discrimination improves attention, reading fluency, and working memory. Frontiers in Human Neuroscience.

[R52] Livingstone M, Hubel D (1988). Segregation of form, color, movement, and depth: anatomy, physiology, and perception. Science.

[R53] Lovegrove WJ, Bowling A, Badcock D, Blackwood M (1980). Specific reading disability: differences in contrast sensitivity as a function of spatial frequency. Science.

[R54] Lui KK, Nunez MD, Cassidy JM, Vandekerckhove J, Cramer SC, Srinivasan R (2021). Timing of readiness potentials reflects a decision-making process in the human brain. Computational Brain & Behavior.

[R55] Manning C, Hassall CD, Hunt LT, Norcia AM, Wagenmakers E-J, Evans NJ, Scerif G (2021b). Behavioural and neural indices of perceptual decision-making in autistic children during visual motion tasks. PsyArXiv.

[R56] Manning C, Kaneshiro B, Kohler PJ, Duta M, Scerif G, Norcia AM (2019). Neural dynamics underlying coherent motion perception in children and adults. Developmental Cognitive Neuroscience.

[R57] Manning C, Tibber MS, Charman T, Dakin SC, Pellicano E (2015). Enhanced integration of motion information in children with autism. Journal of Neuroscience.

[R58] Manning C, Wagenmakers EJ, Norcia AM, Scerif G, Boehm U (2021a). Perceptual decision-making in children: Age-related differences and EEG correlates. Computational Brain & Behavior.

[R59] McKendrick AM, Badcock DR (2004). Motion processing deficits in migraine. Cephalalgia.

[R60] Morey RD, Jeffrey N, Rouder JN (2018). BayesFactor: Computation of Bayes Factors for Common Designs. R package version 0912-42.

[R61] Newsome WT, Paré EB (1988). A selective impairment of motion perception following lesions of the middle temporal visual area (MT). Journal of Neuroscience.

[R62] Nicolson RI, Fawcett AJ (1994). Reaction times and dyslexia. Quarterly Journal of Experimental Psychology A.

[R63] O’Brien G, Yeatman J (2020). Bridging sensory and language theories of dyslexia: toward a multifactorial model. Developmental Science.

[R64] O’Connell RG, Dockree PM, Kelly SP (2012). A supramodal accumulation-to-bound signal that determines perceptual decisions in humans. Nature Neuroscience.

[R65] Olulade OA, Napoliello EM, Eden GF (2013). Abnormal visual motion processing is not a cause of dyslexia. Neuron.

[R66] Perani D, Scifo P, Cicchini GM, Della Rosa P, Banfi C, Mascheretti S (2021). White matter deficits correlate with visual motion perception impairments in dyslexic carriers of the DCDC2 genetic risk variant. Experimental Brain Research.

[R67] Piotrowska B, Willis A (2019). Beyond the global motion deficit hypothesis of developmental dyslexia: A cross-sectional study of visual, cognitive, and socio-economic factors influencing reading ability in children. Vision Research.

[R68] Qian Y, Bi HY (2015). The effect of magnocellular-based visual-motor intervention on Chinese children with developmental dyslexia. Frontiers in Psychology.

[R69] Ratcliff R (1978). A theory of memory retrieval. Psychological Review.

[R70] Raymond JE, Sorensen RE (1998). Visual motion perception in children with dyslexia: Normal detection but abnormal integration. Visual Cognition.

[R71] Shadlen MN, Newsome WT (1996). Motion perception: seeing and deciding. Proceedings of the National Academy of Sciences.

[R72] Shadlen MN, Newsome WT (2001). Neural basis of a perceptual decision in the parietal cortex (area LIP) of the rhesus monkey. Journal of Neurophysiology.

[R73] Skottun BC (2011). On the use of visual motion perception to assess magnocellular integrity. Journal of Integrative Neuroscience.

[R74] Skottun BC (2016). A few remarks on the utility of visual motion perception to assess the integrity of the magnocellular system or the dorsal stream. Cortex.

[R75] Skottun BC, Skoyles JR (2006). Is coherent motion an appropriate test for magnocellular sensitivity?. Brain and Cognition.

[R76] Skottun BC, Skoyles JR (2008). Coherent motion, magnocellular sensitivity and the causation of dyslexia. International Journal of Neuroscience.

[R77] Smith NJ, Kutas M (2015). Regression-based estimation of ERP waveforms: I. The rERP framework. Psychophysiology.

[R78] Snowling MJ, Nash HM, Gooch DC, Hayiou-Thomas ME, Hulme C, Wellcome Language and reading project team (2019a). Developmental outcomes for children at high risk of dyslexia and children with developmental language disorder. Child Development.

[R79] Snowling MJ, Hayiou-Thomas ME, Nash HM, Hulme C (2019b). Dyslexia and developmental language disorder: comorbid disorders with distinct effects on reading comprehension. The Journal of Child Psychology and Psychiatry.

[R80] Sperling AJ, Lu ZL, Manis FR, Seidenberg MS (2006). Motion-perception deficits and reading impairment: it’s the noise, not the motion. Psychological Science.

[R81] Stafford T, Pirrone A, Croucher M, Krystalli A (2020). Quantifying the benefits of using decision models with response time and accuracy data. Behavior Research Methods.

[R82] Stefanac NR, Zhou SH, Spencer-Smith MM, O’Connell R, Bellgrove MA (2021). A neural index of inefficient evidence accumulation in dyslexia underlying slow perceptual decision making. Cortex.

[R83] Stein J (2001). The magnocellular theory of developmental dyslexia. Dyslexia.

[R84] Stein J (2019). The current status of the magnocellular theory of developmental dyslexia. Neuropsychologia.

[R85] Stein J, Walsh V (1997). To see but not to read; the magnocellular theory of dyslexia. Trends in Neurosciences.

[R86] Stone M (1960). Models for choice-reaction time. Psychometrika.

[R87] Talcott JB, Hansen PC, Assoku EL, Stein JF (2000). Visual motion sensitivity in dyslexia: Evidence for temporal and energy integration deficits. Neuropsychologia.

[R88] Ter Braak CJ (2006). A Markov Chain Monte Carlo version of the genetic algorithm Differential Evolution: easy Bayesian computing for real parameter spaces. Statistics and Computing.

[R89] Toffoli L, Scerif G, Snowling MJ, Norcia A, Manning C (2021). Global motion evoked potentials in autistic and dyslexic children: a cross-syndrome approach. Cortex.

[R90] Torgesen JK, Wagner RK, Rashotte CA (2012). Test of Word Reading Efficiency-Second Edition (TOWRE-2).

[R91] Turner BM, Forstmann BU, Wagenmakers EJ, Brown SD, Sederberg PB, Steyvers M (2013). A Bayesian framework for simultaneously modeling neural and behavioral data. NeuroImage.

[R92] Turner BM, Rodriguez CA, Norcia AM, McClure SM, Steyvers M (2016). Why more is better: Simultaneous modeling of EEG, fMRI, and behavioral data. Neuroimage.

[R93] Turner BM, Sederberg PB, Brown SD, Steyvers M (2013). A method for efficiently sampling from distributions with correlated dimensions. Psychological Methods.

[R94] Turner BM, Van Maanen L, Forstmann BU (2015). Informing cognitive abstractions through neuroimaging: the neural drift diffusion model. Psychological Review.

[R95] Ulrich R, Miller J (1994). Effects of truncation on reaction time analysis. Journal of Experimental Psychology: General.

[R96] Vandekerckhove J, Tuerlinckx F, Lee MD (2011). Hierarchical diffusion models for two-choice response times. Psychological Methods.

[R97] Wechsler D (2009). Wechsler Individual Achievement Test.

[R98] Wechsler D (2011). WASI-II: Wechsler abbreviated scale of intelligence.

[R99] Witton C, Talcott JB, Hansen PC, Richardson AJ, Griffiths TD, Rees A, Green GGR (1998). Sensitivity to dynamic auditory and visual stimuli predicts nonword reading ability in both dyslexic and normal readers. Current Biology.

